# Development of a reference data set for assigning *Streptococcus* and *Enterococcus* species based on next generation sequencing of the 16S–23S rRNA region

**DOI:** 10.1186/s13756-019-0622-3

**Published:** 2019-11-15

**Authors:** Maja Kosecka-Strojek, Artur J. Sabat, Viktoria Akkerboom, Anna M. D. (Mirjam) Kooistra-Smid, Jacek Miedzobrodzki, Alexander W. Friedrich

**Affiliations:** 1 Department of Microbiology, Faculty of Biochemistry, Biophysics and Biotechnology, Jagiellonian University, Krakow, Poland; 2 Department of Medical Microbiology, University of Groningen, University Medical Center Groningen, Groningen, The Netherlands; 3 Department of Medical Microbiology, Certe, Groningen, The Netherlands

**Keywords:** *Streptococcus*, *Enterococcus*, NGS, 16S–23S rRNA region, Genetic identification, Diagnostics

## Abstract

**Background:**

Many members of *Streptococcus* and *Enterococcus* genera are clinically relevant opportunistic pathogens warranting accurate and rapid identification for targeted therapy. Currently, the developed method based on next generation sequencing (NGS) of the 16S–23S rRNA region proved to be a rapid, reliable and precise approach for species identification directly from polymicrobial and challenging clinical samples. The introduction of this new method to routine diagnostics is hindered by a lack of the reference sequences for the 16S–23S rRNA region for many bacterial species. The aim of this study was to develop a careful assignment for streptococcal and enterococcal species based on NGS of the 16S–23S rRNA region.

**Methods:**

Thirty two strains recovered from clinical samples and 19 reference strains representing 42 streptococcal species and nine enterococcal species were subjected to bacterial identification by four Sanger-based sequencing methods targeting the genes encoding (i) 16S rRNA, (ii) *sodA*, (iii) *tuf* and (iv) *rpoB*; and NGS of the 16S–23S rRNA region.

**Results:**

This study allowed obtainment and deposition of reference sequences of the 16S–23S rRNA region for 15 streptococcal and 3 enterococcal species followed by enrichment for 27 and 6 species, respectively, for which reference sequences were available in the databases. For *Streptococcus*, NGS of the 16S–23S rRNA region was as discriminative as Sanger sequencing of the *tuf* and *rpoB* genes allowing for an unambiguous identification of 93% of analyzed species. For *Enterococcus*, *sodA*, *tuf* and *rpoB* genes sequencing allowed for identification of all species, while the NGS-based method did not allow for identification of only one enterococcal species. For both genera, the sequence analysis of the 16S rRNA gene was endowed with a low identification potential and was inferior to that of other tested identification methods. Moreover, in case of phylogenetically related species the sequence analysis of only the intergenic spacer region was not sufficient enough to precisely identify *Streptococcus* strains at the species level.

**Conclusions:**

Based on the developed reference dataset, clinically relevant streptococcal and enterococcal species can now be reliably identified by 16S–23S rRNA sequences in samples. This study will be useful for introduction of a novel diagnostic tool, NGS of the 16S–23S rRNA region, which undoubtedly is an improvement for reliable culture-independent species identification directly from polymicrobially constituted clinical samples.

## Background

The genus *Streptococcus* consists of Gram positive bacteria including a numerous clinically significant species which are responsible for wide variety of infections in human and animals with a different manifestation and course [1]. To date, nearly 129 *Streptococcus* and 58 *Enterococcus* species have been identified ([2–4] http://www.bacterio.net/streptococcus.html), but these numbers undergo constant modification. Streptococci are capable to colonize human and animal mucous membranes and considered to be opportunistic pathogens, so in special conditions, they can cause acute infections [5]. Some streptococcal species (e.g. *S. pyogenes* and *S. pneumoniae*) are highly virulent and responsible for severe diseases like pneumonia, necrotizing fasciitis, sepsis and meningitis, the other ones (*S. bovis, S. mutans*, *S. sanguis*, *S. agalactiae* and *S. anginosus*) are involved in a number of clinically relevant diseases like endocarditis, abscesses and other pathological conditions [1, 6, 7]. The genus has undergone considerable taxonomic revisions and, currently based on defined group antigens (A, B, C, E, F, and G) has been divided into different groups: GAS (Group A *Streptococcus*), GBS (Group B *Streptococcus*), group C *Streptococcus*, group G *Streptococcus*, group viridans with subgroups: anginosus, mitis, mutans, salivarius, group bovis [8–10].

Enterococci were initially a part of the *Streptococcus* genus. Currently, they are considered as a separate genus being a part of the human natural microbiota. Enterococcal species are commensals of the gastrointestinal tract of humans and animals and as opportunistic pathogens in immunocompromised patients they can cause acute infections. The *Enterococcus* genus have been reported as the third most common causative agent of bacteremia and infective endocarditis [11–13].

Identification of streptococcal and enterococcal species has been a challenge for decades due to changing taxonomy, names modifications and addition of new species. In routine diagnostic laboratories, phenotypic biochemical methods still play a dominant role. Considering the variability of the strains and species, the differentiation is limited compared to methods based on genetic discrimination and may result in incorrect identification in more than 50% of the cases [14]. The rapidly changing taxonomy also results in a lack of updates in phenotypic databases used in routine diagnostics. If the isolates are not identified at the species level the real impact of single, in particular less frequent species is underreported. Accurate identification is highly desirable for precise therapy, monitoring the spread of infection with epidemiologic characteristics and for investigating the progress of disease [14, 15].

In standard diagnostics, phenotypic tests including automated systems such as Vitek 2 (bioMérieux, La Balme Les Grottes, France) or BD Phoenix (BD Diagnostic Systems, Sparks, MD, USA) as well as the matrix-assisted laser desorption ionization–time of flight mass spectrometry (MALDI-TOF MS) are used for bacteria identification. Especially, commercially available MALDI-TOF MS systems provide accurate identification for many of clinically relevant bacterial species. Nevertheless, the technique so far failed at differentiating between mitis, bovis groups and other closely relative species. Since databases are limited to only some species, further improvements of *Streptococcus* and *Enterococcus* spectra database seem necessary. Moreover, the phenotypic methods are not always reliable enough because of variable expression of phenotypic characteristics [16–18]. The accurate identification at the species level may change the diagnosis and is important to characterize the pathogenic potential of individual species, monitor trends in antimicrobial susceptibility and emerging infections. The ideal method should have a high discriminatory power allowing for identification of closely related species and at the same time should be relatively simple, inexpensive, rapid and reproducible. Therefore, genetic methods based on PCR or sequencing are good candidates for identification purposes. The identification is based on selected nucleic acid target amplification, sequencing and comparison to a reference sequence deposited in a nucleotide database [19, 20].

When polymicrobial samples must be analyzed, it is useful to simultaneously identify species of different genera using a single primer pair. The sequence analysis of the 16S rRNA gene, a highly conserved gene present in all bacteria, can be used for identification at the species level for most bacteria, even those not genetically related, with the same pair of primers [21]. Although this method is widely used and accurate, the high degree of identity of the 16S rRNA gene among the genetically closely related species limits its usefulness for identifying several bacterial species [19, 22, 23].

Next generation sequencing (NGS) has highly improved microbiological genetic investigations by providing a cost-effective way to characterize bacterial genomes. The main advantage of NGS over Sanger sequencing is an ability to produce millions of reads in a single run. Recently, to overcome the limitations of 16S rRNA gene Sanger sequencing, a method based on NGS of the 16S–23S rRNA region has been developed by Sabat and colleagues [24]. This method is based on a PCR amplification of the 16S–23S rRNA region followed by amplicon sequencing on the MiSeq platform (Illumina, Inc., San Diego, CA, USA); the resulting reads are de novo assembled into contigs. Species identification is based on an alignment of the contig sequences with the sequences deposited in the reference databases [24]. This method can be used for identification of common pathogens directly from the patient samples with a high identification potential. This method can also be used for the identification of non-cultured microorganisms, identification of bacterial species in polymicrobial samples or those samples with a too low DNA concentration for direct whole genome sequencing (WGS). However, the main disadvantage of this method is a lack of the 16S–23S rRNA reference sequences for many bacterial species, which hinders the proper interpretation of the results [24]. The main aim of this study was to develop a dataset of reference sequences of the 16S–23S rRNA region for clinically relevant streptococcal and enterococcal species. We also compared the identification potential of NGS-based approach with Sanger sequencing of the 16S rRNA, *sodA, tuf* and *rpoB* genes used for standard streptococci and enterococci identification and determined the cut off values for genus and species level identification.

## Methods

### Bacterial isolates

The bacterial strains used in this study are in detail listed in Table 1. The collection included strains from 42 diverse streptococcal and 9 enterococcal species. Part of the strains are deposited in reference microorganisms collections like the Leibniz Institute DSMZ-German Collection of Microorganisms and Cell Cultures (DSMZ), American Type Culture Collection (ATCC) or Belgian Coordinated Collection of Microorganisms (BCCM). The other strains were clinical isolates from various human and animal sources from Warsaw (National Medicines Institute, Warsaw, Poland), Pescara (Clinical Microbiology and Virology, Spirito Santo Hospital, Pescara, Italy), and Groningen (University Medical Centre Groningen, The Netherlands).

**Table 1:** *Streptococcus* and *Enterococcus* reference species used for analyses

Species	Strain number in reference collection of microorganisms	Species identification based on gene target	Lack of reference sequence in GenBank
*S. acidominimus*	DSM20622	16S rRNA	*sodA, tuf, rpoB*
*S. adjacens*	DSM9848	*tuf, rpoB*	*sodA*
*S. anginosus*	4188/08^b^	16S rRNA*, sodA, tuf, rpoB*	–
*S. australis*	2086/09^b^	*sodA*	*tuf, rpoB*
*S. canis*	S58^a^	*sodA, rpoB*	*tuf*
*S. constellatus*	4093/08^b^	*tuf, rpoB*	*–*
*S. cremoris*	DSM20069	16S rRNA*, sodA, tuf, rpoB*	–
*S. criceti*	DSM20562	16S rRNA*, sodA, tuf, rpoB*	–
*S. cristatus*	3965/07^b^	16S rRNA*, sodA, tuf, rpoB*	–
*S. difficilis*	ATCC700208	16S rRNA*, sodA, tuf, rpoB*	–
*S. downei*	DSM5635	16S rRNA*, sodA, tuf, rpoB*	–
*S. durans*	ATCC19432	*sodA, tuf, rpoB*	–
*S. dysgalactiae*	S59^a^	16S rRNA, *tuf*	*sodA*
*S. equi*	886/14^b^	16S rRNA*, sodA, tuf, rpoB*	–
*S. equinus*	9946/11^b^	16S rRNA	*sodA, tuf, rpoB*
*S. gallolyticus*	S17^a^	16S rRNA, *tuf*	–
*S. gordonii*	381/08^b^	16S rRNA*, sodA, tuf, rpoB*	–
*S. infantarius*	DSM22957	*rpoB*	–
*S. infantis*	3800/09^b^	16S rRNA	*sodA, tuf, rpoB*
*S. intermedius*	1507/09^b^	*sodA, tuf, rpoB*	–
*S. mitis*	PL429^c^	*tuf, rpoB*	–
*S. mutans*	593/09^b^	16S rRNA*, sodA, tuf, rpoB*	–
*S. oligofermentans*	LMG 22279	16S rRNA	*sodA, tuf, rpoB*
*S. oralis*	PL430^c^	*sodA, tuf, rpoB*	–
*S. ovis*	DSM16829	*sodA, rpoB*	*tuf*
*S. parasanguinis*	2605/14^b^	*sodA, tuf, rpoB*	–
*S. pasteurianus*	4035/12^b^	*tuf, rpoB*	*sodA*
*S. pluranimalium*	DSM15636	16S rRNA*, sodA, tuf, rpoB*	–
*S. pneumoniae*	p60^a^	*tuf, rpoB*	–
*S. porcinus*	DSM20725	16S rRNA*, sodA, tuf, rpoB*	–
*S. pseudopneumoniae*	p25^a^	*rpoB*	*tuf*
*S. pseudoporcinus*	DSM18513	16S rRNA, *rpoB*	*sodA, tuf*
*S. pyogenes*	S43^a^	16S rRNA, *tuf, rpoB*	–
*S. saccharolyticus*	ATCC43076	16S rRNA*, sodA, tuf, rpoB*	–
*S. salivarius*	3917/16^b^	*sodA, tuf, rpoB*	–
*S. sanguinis*	4416/10^b^	16S rRNA*, sodA, tuf, rpoB*	–
*S. sinensis*	DSM14990	16S rRNA*, sodA, tuf, rpoB*	–
*S. sobrinus*	864/02^b^	16S rRNA*, sodA, tuf, rpoB*	–
*S. suis*	174/12^b^	16S rRNA*, sodA, tuf, rpoB*	–
*S. tigurinus*	ATCC15914	*sodA, tuf, rpoB*	–
*S. uberis*	DSM20569	*sodA, tuf, rpoB*	–
*S. urinalis*	PL432^c^	16S rRNA*, sodA, tuf, rpoB*	–
*E. avium*	E16^a^	*sodA, tuf, rpoB*	–
*E. casseliflavus*	E1^a^	*sodA, tuf, rpoB*	–
*E. cecorum*	DSM20682	16S rRNA*, sodA, tuf, rpoB*	–
*E. durans*	E4^a^	*sodA, tuf, rpoB*	–
*E. faecium*	E18^a^	*tuf, rpoB*	*sodA*
*E. faecalis*	E12^a^	*sodA, tuf, rpoB*	–
*E. hirae*	E9^a^	*sodA, tuf, rpoB*	–
*E. porcinus*	LMG 12287	16S rRNA, *sodA, rpoB*	*tuf*
*E. raffinosus*	E11^a^	*sodA, tuf, rpoB*	–

^a^clinical isolate; Pescara, Italy

^b^clinical isolate; Warsaw, Poland

^c^clinical isolate; University Medical Center Groningen, The Netherlands

### Genomic DNA extraction

For genomic DNA extraction, the isolates were grown for 18–20 h at 37 °C on blood agar plates in microaerophilic conditions or with 5% CO_2_. Two strains, *S. cremoris* (DSM20069) and *S. difficilis* (ATCC700208) were grown at 30 °C. A full inoculation loop of 10 μl of bacterial colonies was homogenized with a TissueLyser II (Qiagen, Germantown, MD, USA). Total DNA was extracted by enzymatic lysis using the buffers and solutions provided with the DNeasy Blood and Tissue Kit (Qiagen, Germantown, MD, USA) according to manufacturer’s instructions. To obtain an accurate quantification of the extracted genomic DNA for NGS, a fluorometric method specific for duplex DNA, a Qubit dsDNA BR Assay Kit and a Qubit fluorometer 2.0 (Life Technologies, Inc., Eggenstein, Germany) were used according to the manufacturer’s instructions.

### PCR amplification and Sanger sequencing of 16S rRNA, *sodA*, *tuf* and *rpoB* genes

All reference strains were identified at the species level by polymerase chain reaction (PCR) and Sanger sequencing of 16S rRNA, *sodA*, *tuf* and *rpoB* genes. The 16S rRNA gene was amplified using the primers LPW57 (5′-AGTTTGATCCTGGCTCAG-3′) and LPW58 (5′-AGGCCCGGGAACGTATTCAC-3′) as previously described [25]. The PCR program was as follow: initial denaturation for 2 min at 94 °C, then followed by 25 cycles of denaturation at 94 °C for 30 s, annealing at 58 °C for 30 s and extension at 72 °C for 60 s. The final extension was for 5 min at 72 °C.

For the *sodA* gene, the internal fragment which represents 83% of the gene (430 bp), was amplified with the primers d1 (5′-CCITAYICITAYGAYGCIYTIGARCC-3′) and d2 (5′-ARRTARTAIGCRTGYTCCCAIACRTC-3′) as previously described [26]. The PCR mixtures were initially denatured for 3 min at 95 °C and then followed by 35 cycles of denaturation at 95 °C for 30 s, annealing at 40 °C for 60 s, extension at 72 °C for 90 s with final extension at 72 °C for 10 min. For some strains, the PCR product was not specific in these conditions and the annealing temperature was increased to 43 °C, 46 °C or 50 °C. For strain DSM9848 (*S. adjacens*) the aforementioned primers did not yield any amplification product and primers sodA-F (5′- TRCAYCATGAYAARCACCAT-3′) and sodA-R (5′- ARRTARTAMGCRTGYTCCCARACRTC-3′) were used [19]. Amplification of the DNA fragments was performed with predenaturation for 5 min at 94 °C followed by 30 cycles of denaturation at 94 °C for 30 s, annealing at 45 °C for 60 s, extension at 72 °C for 30 s with final extension at 72 °C for 5 min.

For *tuf*, an 830-bp portion of the gene, was amplified with the primers Tuf-F (5′-CCAATGCCACAAACTCGT-3′) and Tuf-R (5′-CCTGAACCAACAGTACGT-3′) as previously described [20]. The PCR program was as follow: initial denaturation for 2 min at 95 °C and then followed by 30 cycles of denaturation at 94 °C for 30 s, annealing at 50 °C for 30 s, extension at 72 °C for 90 s with final extension at 72 °C for 10 min. For some strains, the PCR product was not specific in these conditions and the annealing temperature was increased to 53 °C, 56 °C or 59 °C. For strain LMG 12287 (*E. porcinus*) the aforementioned primers did not yield any amplification product and primers U1 (5′-AAYATGATIACIGGIGCIGCICARATGGA-3′) and U2 (5′- AYRTTITCICCIGGCATIACCAT-3′) were used [27]. Amplification of the DNA fragments was performed with predenaturation for 3 min at 95 °C followed by 35 cycles of denaturation at 95 °C for 30 s, annealing at 55 °C for 30 s, extension at 72 °C for 60 s with final extension at 72 °C for 7 min.

The partial *rpoB* gene (740 bp) was amplified with the primers Strepto F (5′- AARYTIGGMCCTGAAGAAAT-3′) and Strepto R (5′- TGIARTTTRTCATCAACCATGTG − 3′) as previously described [28] with slight modifications in the PCR program: initial denaturation for 2 min at 95 °C and then followed by 35 cycles of denaturation at 94 °C for 30 s, annealing at 52 °C for 30 s, extension at 72 °C for 60 s with final extension at 72 °C for 5 min. For some strains, the PCR product was not specific in these conditions and the annealing temperature was increased to 55 °C.

All PCR products were resolved by electrophoresis using a 2200 TapeStation System (Agilent Technologies, Santa Clara, CA, USA) and then purified using the DNA Clean & Concentrator™-5 purification kit (Zymo Research, Irvine, CA, USA).

For the Sanger sequencing of 16S rRNA, *sodA*, *tuf* and *rpoB* genes, the same primers as for PCR amplification were used. For the 16S rRNA, *tuf* and *rpoB* genes a total amount of 200 ng of PCR product was sequenced and for the *sodA* gene 100 ng.

### Next generation sequencing of the 16S–23S rRNA region

Amplification of the 16S–23S rRNA region was performed using primer 16S-27F (5′-AGAGTTTGATCMTGGCTCAG-3′) and primer 23S-2490R (5′-GACATCGAGGTGCCAAAC-3′) as described previously [24]. The PCR program was as follow: initial denaturation for 2 min at 94 °C and then followed by 30 cycles of denaturation at 94 °C for 30 s, annealing at 66 °C for 30 s, extension at 72 °C for 120 s with final extension at 72 °C for 5 min. The obtained PCR products were purified and the DNA libraries were prepared with Nextera XT DNA Sample Preparation Kit (Illumina) according to the manufacturer’s instructions. The indexed libraries were pooled and loaded onto an Illumina MiSeq reagent cartridge using MiSeq reagent kit v3 and 600 cycles. The 2 × 300 bp sequencing was run on an Illumina MiSeq platform.

### Data analysis

The Sanger sequencing results were analyzed using the Chromas (v. 2.6.2.; Technelysium Pty Ltd., South Brisbane, Australia) software. The obtained sequences were analyzed using nucleotide BLAST (Basic Local Alignment Search Tool, http://www.ncbi.nlm.nih.gov/BLAST/) and aligned to the reference sequences deposited in the GenBank (https://www.ncbi.nlm.nih.gov/nucleotide/) and leBIBI (https://umr5558-bibiserv.univ-lyon1.fr/lebibi/lebibi.cgi) databases. The best and the second best species alignment were analyzed. According to the criteria developed by Sabat et al. in 2017 [24], the bacterial species were assigned when the identity score was 99% or higher and the identity score differences with the next closest species was ≥0.2%. Therefore, the identification at the species level using Sanger sequencing of the 16S rRNA (1284-bp), *sodA* (430-bp), *tuf* (830-bp) and *rpoB* (740-bp) gene fragments was considered as unambiguous for sequences different in at least 3, 2, 3 and 3 nucleotides, respectively. The identification at the species level using NGS of the whole 16S–23S rRNA region (4.3-kb), 16S rRNA gene (1.5-kb), intergenic region (330-bp) and 23S rRNA gene (2.5-kb) was considered as unambiguous for sequences different in at least 9, 3, 2 and 5 nucleotides, respectively. The sequences were aligned in ClustalW [29] and the phylogenetic trees were constructed using the Neighbor-Joining method [30–32]. The tree topologies were compared using Compare2Trees program [33]. The pairwise comparison of each pair of sequences was obtained using CLC Genomics Workbench (v. 8.1; Qiagen, Germantown, MD, USA) considering deletions as differences.

NGS generated 35,000–350,000 sequencing reads for pure culture to obtain a minimum coverage of 1000 per sample. The fastq files (Illumina MiSeq) with read length of 300 nucleotides were de novo assembled with the DNASTAR SeqMan NGen software (v. 15.3; DNASTAR, Madison, WI, USA). During read assembly, reads shorter than 250 nucleotides were excluded. The minimum match percentage was 85% or 93% and the mer size was set as 31 nucleotides. After assembly, mean sample coverage was 6680.50-fold. However, the coverage per sample varied between 1983.38- and 23,643.77-fold. Only runs with a Q30 read quality score of > 80% were accepted. To further determine sequencing errors of the Illumina MiSeq platform three types of errors were investigated: insertion, deletion and mismatch. If a single nucleotide polymorphism (SNP) variant was identified in the consensus sequence, it was at maximum level of 5.36% with 932-fold coverage. Such SNP values were regarded as the potential sequencing errors and discarded from further analysis. If the assembly resulted in multiple contigs, the obtained ones were checked for length and quality in order to select the longest main contig with the highest reads amount assigned. Finally, the main contig was exported as fasta file for use in the subsequent analyses. For all species the main contig comprising the whole 16S–23S rRNA region, counting for *Streptococcus* from 4251 (*S. adjacens*) to 4732 nucleotides (*S. equinus*) and for *Enterococcus* from 4224 nucleotides (*E. cecorum*) to 4381 nucleotides (*E. faecium*), was obtained. Species identification was based on alignment of contig sequences with 16S–23S rRNA sequences deposited in the GenBank database using nucleotide BLAST and also compared to leBIBI database (the 16S rRNA gene sequence as reference).

### Nucleotide sequence accession numbers

The 255 sequences for 42 *Streptococcus* and 9 *Enterococcus* species were annotated using the NCBI BankIt tool and deposited in the GenBank database (http://www.ncbi.nlm.nih.gov/genbank/) under accession numbers: for the 16S–23S rRNA region, MK330555-MK330596 and MK322658-MK322666; for the 16S rRNA gene, MK330513-MK330554 and MK322649-MK322657; for the *sodA* gene, MK322556-MK322597 and MK308717-MK308725; for the *tuf* gene, MK322607-MK322648 and MK322598-MK322606; and for the *rpoB* gene, MK322514-MK322555 and MK308708-MK308716. The NGS of 16S–23S rRNA region raw reads were deposited in the European Nucleotide Archive (ENA) (https://www.ebi.ac.uk/ena) under study accession number: PRJEB32803 (ERP115525).

## Results

### Identification potential of Sanger sequencing methods for *Streptococcus* and *Enterococcus* species

All strains from the collection were characterized by Sanger sequencing of the 16S rRNA, *sodA*, *tuf* and *rpoB* genes. The identification to the species level was not possible by all targets used due to identical or almost identical sequence (Table 2 and Additional file 1: Tables S1-S8) or the lack of some reference sequences in the GenBank (v. 231.0; June 21, 2019) database (Table 1). Therefore, the identification was confirmed by 1 target for 7 streptococcal species, by 2 targets for 10 streptococcal and 1 enterococcal species, by 3 targets for 8 streptococcal and 7 enterococcal species and by 4 targets for the vast majority of the species (17 *Streptococcus* and 1 *Enterococcus* species) (Table 1). The reference sequences for all *Streptococcus* and *Enterococcus* species were available only for 16S rRNA gene.

**Table 2:** The comparison of indistinguishable pairs or groups of *Streptococcus* and *Enterococcus* species after Sanger sequencing of 16S rRNA, *sodA*, *tuf* and *rpoB* genes and NGS of 16S rRNA, 23S rRNA genes, intergenic spacer region and whole 16S–23S rRNA region

Sequencing target	Indistinguishable pairs or groups of *Streptococcus* species	Indistinguishable pairs or groups of *Enterococcus* species
Sanger 16S rRNA gene	*S. adjacens - Granulicatella para-adiacens; S. australis - S. mitis; S. canis - S. dysgalactiae; S. constellatus - S. anginosus; S. durans - E. hirae; S. infantarius - S. equinus; S. intermedius - S. anginosus; S. mitis - S. pneumoniae - S. pseudopneumoniae; S. oralis - S. sanguinis; S. ovis - S. minor; S. parasanguinis - S. mitis; S. pasteurianus - S. gallolyticus; S. salivarius - S. equinus; S. tigurinus - S. mitis; S. uberis - S. hongkongensis*	*E. avium - E. gilvus; E. casseliflavus - E. gallinarum; E. durans - E. faecium; E. faecalis - Weissella cibaria; E. hirae - E. faecium; E. raffinosus - E. gilvus*
Sanger *sodA* gene	*S. constellatus - S. anginosus; S. dysgalactiae - S. pyogenes; S. gallolyticus - S. bovis; S. infantarius - S. equinus; S. mitis - S. pneumoniae - S. pseudopneumoniae; S. pyogenes - S. equisimilis*	*–*
Sanger *tuf* gene	*S. infantis - S. tigurinus; S. infantarius - S. equinus*	*–*
Sanger *rpoB* gene	*S. dysgalactiae - S. pyogenes; S. gallolyticus - S. pasteurianus*	*–*
NGS 16S rRNA gene	*S. australis - S. oralis; S. canis - S. dysgalactiae; S. constellatus - S. milleri; S. durans - E. hirae; S. infantarius - S. equinus; S. intermedius - S. anginosus; S. mitis - S. pneumoniae - S. pseudopneumoniae; S. ovis - S. minor; S. parasanguinis - Okadaella gastrococcus; S. pasteurianus - S. gallolyticus; S. salivarius - S. equinus; S. tigurinus - S. mitis; S. uberis - S. hongkongensis*	*E. avium - E. gilvus; E. casseliflavus - E. gallinarum; E. durans - E. faecium; E. faecalis - Weissella cibaria; E. hirae - E. durans; E. raffinosus - E. gilvus*
NGS intergenic spacer region	*S. constellatus - S. milleri; S. infantarius - S. equinus; S. infantis - S. pneumoniae; S. mitis - S. oralis - S. pneumoniae - S. pseudopneumoniae; S. salivarius - S. equinus; S. tigurinus - S. infantis*	*E. casseliflavus - E. gallinarum*
NGS 23S rRNA gene	*S. anginosus - S. milleri; S. constellatus - S. milleri; S. cremoris - Lactococcus lactis; S. infantarius - S. equinus; S. mitis - S. pneumoniae - S. pseudopneumoniae; S. salivarius - S. equinus; S. tigurinus - S. mitis*	*E. casseliflavus - E. gallinarum; E. hirae - E. durans*
NGS 16S–23S rRNA region	*S. infantarius - S. equinus; S. pseudopneumoniae - S. pneumoniae; S. tigurinus - S. oralis*	*E. casseliflavus - E. gallinarum*

### Sequence analysis of the 16S–23S rRNA region

The sequence analysis of the 16S–23S rRNA region was performed on 51 strains from our collection representing 42 *Streptococcus* and 9 *Enterococcus* species. Search of the GenBank database showed that the sequences for the 16S–23S rRNA region were available for 27 *Streptococcus* species and 6 *Enterococcus* species, while this study allowed for the obtainment and deposition of nucleotide sequences for the additional 15 and 3 species, respectively. Taking into consideration the differences in length of an intergenic spacer located between the 16S and 23S rRNA genes, the average sequence length of the 16S–23S rRNA region was determined and equaled 4346 nucleotides for *Streptococcus* and 4299 for *Enterococcus*. The highest identity of 16S–23S rRNA region among *Streptococcus* species was found between *S. infantis* and *S. tigurinus* showing 99.7% sequence homology (13 nucleotides of difference), while the highest nucleotide difference was found between *S. adjacens* and *S. criceti* and equaled 1209 nucleotides (74.4% identity). For *Enterococcus*, the highest identity was found between *E. avium* and *E. raffinosus* showing 98.6% sequence homology (62 nucleotides of difference). The highest nucleotide difference was found between *E. cecorum* and *E. hirae* and equaled 431 nucleotides (90.1% identity) (Additional file 1: Tables S9 and S10). We also determined the lengths of 16S rRNA gene, intergenic spacer region and 23S rRNA gene for all species used in this study (Additional file 1: Table S11).

To show the relationships between species, the phylogenetic trees were constructed. The pairwise overall topological scores computed by Compare2Trees based on *Streptococcus* 16S rRNA, *rpoB*, *sodA*, *tuf*, and 16S–23S rRNA sequences ranged from 61.7 to 72.4% (Additional file 1: Figure S1). For *Enterococcus*, the distances between two trees in terms of topology were more diverse, reaching the lowest and highest values, 56.4 and 80.6%, respectively. All targets showed *S. cremoris* and group of species: *S. adjacens, S. durans* and *S. saccharolyticus* are distantly related to other species. For *Enterococcus* species the *E. cecorum* was distantly related to other species. The analysis of the phylogenetic tree of the 16S–23S rRNA region showed similar clustering as in dendrogram based on 16S rRNA gene sequencing, but more discriminative with unambiguous identification for all species (Additional file 1: Figure S1).

### Criteria for assigning *Streptococcus* and *Enterococcus* at the species level

We performed the BLAST analysis based on alignment of the 16S–23S rRNA sequences obtained during the current study with those deposited in GanBank (Table 3) using criteria proposed by Sabat et al. [24]. For the assignment at the species level, we used identity score > 99% and differences with the next closest species at ≥0.2%, which reflected the difference of at least 9 nucleotides by sequencing the 16S–23S rRNA region. In comparison to sequences already deposited in GenBank, for a great majority of species (*Streptococcus*, *n* = 39, *Enterococcus*, *n* = 8) those criteria allowed the NGS-based approach the proper identification, except *S. australis* with a first identification score at 97.4%. For next 4 species (*Streptococcus*, *n* = 3, *Enterococcus*, *n* = 1), the first criterium of > 99% identity was fulfilled but the differences with the next closest species ranged from 2 to 7 nucleotides so the species could not be unambiguously assigned.

**Table 3:** The *Streptococcus* and *Enterococcus* species alignment of 16S–23S rRNA region to GenBank^a, b^

Species	NGS of 16S -23S rRNA region BLAST GenBank 1st ID	Score	NGS of 16S - 23S rRNA region BLAST GenBank 2nd ID	Score	Difference between 1st and 2nd ID
*Streptococcus anginosus*	*Streptococcus anginosus*	4410/4411 (99.9%)	*Streptococcus intermedius*	4287/4419 (97.0%)	2.9%
*Streptococcus australis*	*Streptococcus australis*	**4159/4270 (97.4%)**	*Streptococcus oralis*	4204/4255 (98.8%)	1.4%
*Streptococcus canis*	*Streptococcus canis*	4296/4301 (99.9%)	*Streptococcus dysgalactiae*	4206/4303 (97.8%)	2.%
*Streptococcus constellatus*	*Streptococcus constellatus*	4324/4340 (99.6%)	*–*	–	–
*Streptococcus cremoris*	*Streptococcus cremoris*	4317/4317 (100%)	*Lactococcus garvieae*	4043/4391 (92.1%)	7.9%
*Streptococcus criceti*	*Streptococcus criceti*	4649/4649 (100%)	*–*	–	–
*Streptococcus durans*	*Streptococcus durans*	4359/4366 (99.8%)	*Enterococcus hirae*	4330/4368 (99.1%)	0.7%
*Streptococcus dysgalactiae*	*Streptococcus dysgalactiae*	4300/4301 (99.9%)	*Streptococcus canis*	4201/4303 (97.6%)	2.3%
*Streptococcus equi*	*Streptococcus equi*	4429/4429 (100%)	*–*	–	–
*Streptococcus gallolyticus*	*Streptococcus gallolyticus*	4278/4284 (99.9%)	*Streptococcus pasteurianus*	4259/4286 (99.4%)	0.5%
*Streptococcus gordonii*	*Streptococcus gordonii*	4265/4267 (99.9%)	*Streptococcus sanguinis*	4161/4291 (97.0%)	2.9%
*Streptococcus infantarius*	*Streptococcus infantarius*	4282/4284 (99.9%)	*Streptococcus equinus*	4284/4284 (100%)	**0.1%**
*Streptococcus intermedius*	*Streptococcus intermedius*	4284/4291 (99.8%)	*Streptococcus gordonii*	4138/4302 (96.2%)	3.6%
*Streptococcus mitis*	*Streptococcus mitis*	4247/4259 (99.7%)	*Streptococcus pneumoniae*	4238/4259 (99.5%)	0.2%
*Streptococcus mutans*	*Streptococcus mutans*	4408/4408 (100%)	*–*	–	–
*Streptococcus oligofermentans*	*Streptococcus oligofermentans*	4263/4263 (100%)	*Streptococcus sanguinis*	4145/4285 (96.7%)	3.3%
*Streptococcus oralis*	*Streptococcus oralis*	4251/4259 (99.8%)	*Streptococcus pneumoniae*	4237/4257 (99.5%)	0.3%
*Streptococcus parasanguinis*	*Streptococcus parasanguinis*	4227/4261 (99.2%)	*Streptococcus sanguinis*	4137/4273 (97.6%)	1.6%
*Streptococcus pasteurianus*	*Streptococcus pasteurianus*	4283/4284 (99.9%)	*Streptococcus gallolyticus*	4258/4286 (99.4%)	0.5%
*Streptococcus pluranimalium*	*Streptococcus pluranimalium*	4295/4304 (99.8%)	*Streptococcus halotolerans*	4163/4316 (96.5%)	3.3%
*Streptococcus pneumoniae*	*Streptococcus pneumoniae*	4255/4258 (99.9%)	*Streptococcus pseudopneumoniae*	4238/4259 (99.5%)	0.4%
*Streptococcus porcinus*	*Streptococcus porcinus*	4558/4558 (100%)	*–*	–	–
*Streptococcus pseudopneumoniae*	*Streptococcus pseudopneumoniae*	4245/4261 (99.6%)	*Streptococcus pneumoniae*	4243/4260 (99.6%)	**0.0%**
*Streptococcus pseudoporcinus*	*Streptococcus pseudoporcinus*	4498/4498 (100%)	*–*	–	–
*Streptococcus pyogenes*	*Streptococcus pyogenes*	4435/4436 (99.9%)	*Streptococcus suis*	4199/4457 (94.2%)	5.7%
*Streptococcus salivarius*	*Streptococcus salivarius*	4283/4283 (100%)	*Streptococcus vestibularis*	4270/4283 (99.7%)	0.3%
*Streptococcus sanguinis*	*Streptococcus sanguinis*	4254/4275 (99.5%)	*Streptococcus gordonii*	4162/4292 (97.0%)	2.5%
*Streptococcus sobrinus*	*Streptococcus sobrinus*	4412/4424 (99.7%)	*–*	–	–
*Streptococcus suis*	*Streptococcus suis*	4420/4420 (100%)	*–*	–	–
*Streptococcus tigurinus*	*Streptococcus oralis subsp. tigurinus*	4223/4261 (99.1%)	*Streptococcus oralis*	4221/4261 (99.1%)	**0.0%**
*Streptococcus uberis*	*Streptococcus uberis*	4352/4352 (100%)	*Streptococcus iniae*	4197/4358 (96.3%)	3.7%
*Streptococcus urinalis*	*Streptococcus urinalis*	4291/4292 (99.9%)	*Streptococcus agalactiae*	4136/4305 (96.1%)	3.8%
*Enterococcus casseliflavus*	*Enterococcus casseliflavus*	4263/4266 (99.9%)	*Enterococcus gallinarum*	4256/4266 (99.8%)	**0.1%**
*Enterococcus cecorum*	*Enterococcus cecorum*	4214/4224 (99.8%)	*Lactobacillus plantarum*	3754/4277 (88.0%)	11.8%
*Enterococcus durans*	*Enterococcus durans*	4311/4313 (99.9%)	*Enterococcus silesiacus*	4083/4342 (94.0%)	5.9%
*Enterococcus faecium*	*Enterococcus faecium*	4377/4381 (99.9%)	*Enterococcus hirae*	4321/4387 (98.5%)	1.4%
*Enterococcus faecalis*	*Enterococcus faecalis*	4258/4262 (99.9%)	*Enterococcus wangshanyuanii*	4122/4270 (96.5%)	3.4%
*Enterococcus hirae*	*Enterococcus hirae*	4359/4367 (99.8%)	*Enterococcus durans*	4330/4368 (99.1%)	0.7%

^a^For species not included in a Table (*Streptococcus acidominimus, Streptococcus adjacens, Streptococcus cristatus, Streptococcus difficilis, Streptococcus downei, Streptococcus equinus, Streptococcus infantis, Streptococcus ovis, Streptococcus saccharolyticus, Streptococcus sinensis, Enterococcus avium, Enterococcus porcinus, Enterococcus raffinosus*), there are no reference genomes available. ^b^Species for which the NGS-based approach did not allow the proper identification based on previously described criteria are indicated in bold

### Intraspecies nucleotide sequence variation of the 16S–23S RNA region

To show the variability of 16S–23S rRNA region, the nucleotide sequence variation within *Streptococcus* and *Enterococcus* species was determined (Additional file 1: Table S12). The analysis was performed for those species for which at least one nucleotide sequence of the 16S–23S rRNA region could be found in the GenBank database. For almost all species, the length of the 16S–23S rRNA region was the same within a species when the sequences obtained in this study and those deposited in GenBank were compared. The length of 16S–23S rRNA region was different within the same species only in case of *S. acidominimus* and *S. equinus*. The nucleotide variation within *Streptococcus* species accounted from 0.07 to 2.74%, with the exception of *S. pneumoniae* for which the intraspecies nucleotide variation was 11.65%. For *Enterococcus* species, the nucleotide variation accounted from 0.02 to 2.67%.

### Comparison of identification potential of NGS of the 16S–23S rRNA region to the methods based on Sanger sequencing

For *Streptococcus* species, NGS of the 16S–23S rRNA region, *tuf* and *rpoB* genes Sanger sequencing had the highest identification potential allowing for an unambiguous identification of 93% of analyzed species (Table 4). For *Enterococcus* species, *sodA*, *tuf* and *rpoB* genes sequencing allowed for identification of all species, while the NGS-based method did not allow for identification of only one enterococcal species (Table 5). For both genera 16S rRNA gene Sanger sequencing had the lowest identification potential of all the methods used.

**Table 4:** Summary of the species identification, nucleotide differences range and amount of available reference sequences based on 16S rRNA, *sodA*, *tuf* and *rpoB* genes and 16S–23S rRNA region for *Streptococcus* genus

	16S rRNA gene	*sodA* gene	*tuf* gene	*rpoB* gene	NGS 16S–23S rRNA
Unambiguous species identification	25 species (60%)	34 species (81%)	39 species (93%)	39 species (93%)	39 species (93%)
The lowest amount of nucleotides differences	0	0	0	0	2
The highest amount of nucleotides differences	228	176	205	186	1209
No. of species without reference sequences in the databases	0	8	9	5	15

**Table 5:** Summary of the species identification, nucleotide differences range and amount of available reference sequences based on 16S rRNA, *sodA*, *tuf* and *rpoB* genes and 16S–23S rRNA region for *Enterococcus* genus

	16S rRNA gene	*sodA* gene	*tuf* gene	*rpoB* gene	NGS 16S–23S rRNA
Unambiguous species identification	2 species (22%)	9 species (100%)	9 species (100%)	9 species (100%)	8 species (89%)
The lowest amount of nucleotides differences	0	5	10	5	7
The highest amount of nucleotides differences	90	124	114	118	431
No. of species without reference sequences in the databases	0	1	1	0	3

### The identification potential of 16S rRNA, 23S rRNA genes, intergenic spacer region and 16S–23S rRNA region

We also determined the identification potential of each part of the 16S–23S rRNA region separately. Each fragment alone showed a drop in identification potential for *Streptococcus* species in comparison to the whole region (Table 2). The rates of identification to the species level using sequences of the 16S rRNA gene, intergenic region, 23S rRNA gene and whole 16S–23S rRNA region were 64, 71, 86 and 93%, respectively. In case of *Enterococcus*, the species identification potential of the intergenic spacer region was as good as the whole region and equaled 89%, and superior to that of the 16S rRNA and 23S rRNA genes, 33 and 78%, respectively.

## Discussion

Because of the clinical significance and challenging taxonomy changes of *Streptococcus* and *Enterococcus* species, an accurate identification at the species level is highly desirable to permit a more precise determination of host-pathogen relationships and to better understand pathogenic potential of various streptococcal and enterococcal species. Phenotypic identification of streptococcal and enterococcal species appears to be unsatisfactory, unreliable, and irreproducible [14, 16, 18]. This is a reason for applying genetic methods in standard microbiological diagnostics. If an unknown organism needs to be identified in a clinical sample, 16S rRNA gene sequencing is the method of choice because of the availability of universal primers [34]. The 16S rRNA gene sequencing is an excellent target for most streptococcal and enterococcal species but the differentiation between the species is difficult due to the insufficient heterogeneity within the 16S rRNA gene. Most of the reports show that the discriminatory power of 16S rRNA gene sequencing is very low for closely related *Streptococcus* and *Enterococcus* species [1, 20, 35–37]. Moreover, some authors claim that accuracy of identification of bacterial species with 16S rRNA gene sequencing is limited by the low quality of the sequences deposited in publicly available databases [38]. The other targeted sequencing methods do have a higher identification potential than 16S rRNA gene sequencing but are limited to only genetically related genera [20].

Within this study, we used a combination of four genetic targets (16S rRNA, *sodA*, *tuf* and *rpoB*) in order to unambiguously confirm the identification at the species level for all *Streptococcus* and *Enterococcus* strains tested. The analysis based on only one gene is not recommended because of possible gene duplication, lateral gene transfer or gene loss, which can distort the results [39]. The Compare2Trees data showed that the topology of phylogenetic trees obtained in this study was not very similar. These findings indicated that the genes, even highly conserved rRNA genes, are subject to recombination and that these events may render species identification challenging.

This study showed that NGS of the 16S–23S rRNA region was as discriminative as *tuf* and *rpoB* genes sequencing for *Streptococcus* species. In case of *Enterococcus*, *sodA*, *tuf* and *rpoB* genes sequencing allowed for identification of all species, while the NGS-based method did not allow for identification of only *E. casseliflavus*. Moreover, NGS of the 16S–23S rRNA region showed the same clustering like other methods. As NGS of the 16S–23S rRNA region uses universal primers it is applicable to different genetically unrelated bacterial genera [24].

The purpose of this study was not only to compare five sequence-based methods for streptococci and enterococci identification but primarily to develop streptococcal and enterococcal reference sequence datasets of the 16S–23S rRNA region. NGS of the 16S–23S rRNA region developed by Sabat and colleagues [24] provides the ability to detect microorganisms not only in samples from mixed polymicrobial colonization and infections consisting commensal microorganisms and the whole persistant microbiome. However, this method currently suffers from a lack of reference sequences in the GenBank database for many bacterial species. Before this study the 16S–23S rRNA sequences were available for 27 clinically relevant *Streptococcus* and 6 *Enterococcus* species, respectively. Our investigations allowed obtainment and deposition of the 16S–23S rRNA sequences for the next 15 streptococcal and 3 enterococcal species making identification of *Streptococcus* and *Enterococcus* species feasible. Moreover, we determined that in case of phylogenetically related species, like mitis group, the analysis of only the intergenic spacer region are not sufficient enough to precisely identify *Streptococcus* strains at the species level.

In order to identify strains at the species level, the reference sequence with the highest identity score needs to be found. For several *Streptococcus* and *Enterococcus* species, only one or a few reference 16S–23S rRNA sequences can be found during BLAST searches in the GenBank database. In such cases, it is possible that the sequence obtained during a study belongs to a different evolutionary cluster within a species than the reference and the nucleotide differences between them are high (more than 1%). Then, it is not possible to assign bacterial species with the identity score 99% or higher. During the current study, such instance was found only for *S. australis*. If more reference sequences are deposited in the genetic sequence databases, representing evolutionary diverse lineages, species will always be assigned with an identity score above 99%.

NGS of 16S–23S rRNA approach proved to be an excellent tool for identification at the species level for a great majority of *Streptococcus* and *Enterococcus* strains. Although, there were some problematic cases especially in bovis and mitis groups as the groups have undergone several reclassifications. *S. infantarius* was alternately classified as *S. lutetiensis* or *S. infantarius*, finally described as the second one [40]. Moreover, this species is a part of *S. bovis*/*S. equinus* complex and therefore challenging to be properly identified [41, 42]. In our study, in case of *S. infantarius*, the next closest species was *S. equinus* with an alignment to only one and not published genome assembly. Similar situation was for *S. tigurinus* which at first was a subspecies, then a separate species and in 2016 again proposed to be classified as *S. oralis* subsp. *tigurinus* [43, 44]. As showed in results, our sequence was aligned to *S. oralis* subsp. *tigurinus* (4223/4261) and the next closest species was the sequence of unpublished *S. oralis* (4221/4261). For both *S. mitis* and *S. pseudopneumoniae*, the next best alignment was to *S. pneumoniae*. As the problems in accurate dentification of mitis group are described [45, 46], we believe that the increase of deposited sequences for *S. mitis* and *S. pseudopneumoniae* will allow for an unequivocal identification. It is very important to develop a well-curated database with a verification of deposited sequences in terms of proper organism identification. For now, the sequences that are not published should not be considered as reference ones. There is no previous single study with a same dataset of reference sequences for genes commonly used for streptococci and enterococci identification, so usually those sequences cannot be compared. In this study, we have not only deposited such dataset for 4 commonly used identification targets but also added a package of sequences for a new identification tool with a high identification potential.

As the NGS-based techniques allow culture free detection of a theoretically unlimited number of pathogens it is necessary to precisely identify the species. Concerning the opportunistic pathogens and those not dominating in a sample, the accurate identification indicates the correct identification of an etiological factor of the infection. Since the benchtop sequencers were introduced, the NGS is likely to become a diagnostic tool in microbiological laboratories [47]. The NGS of 16S–23S rRNA region was developed to fill the gap between the conventional methods (culture and PCR) and metagenomics but as highlighted by Sabat et al. still suffers for the lack of reference sequences for many bacterial species. The development of *Streptococcus* and *Enterococcus* 16S–23S rRNA sequences dataset is a first step to come across this limitation. We are currently working on development of datasets for next clinically relevant genera.

The PCR-based methods as a tool for microbial identification, are superior to NGS-based methods in cost and speed. Although, when unknown bacteria needs to be identified, it is challenging to accurately choose the appropriate method as targets such as *sodA*, *tuf* or *rpoB* sequencing are genus-specific. The reagents and consumables costs for PCR-based methods combined with Sanger sequencing amount to ~ 10 € per sample and in a turnaround time of 2 days. The costs may be higher if the first choice of the method is not correct and those methods can be applied only for pure cultures. Year by year, the NGS techniques become cheaper and currently, the total costs of all reagents and consumables for NGS of 16S–23S rRNA region amount to ~ 150 € per sample with a turnaround time of 6–8 days. With the NGS-based approach the whole species content can be detected within one sequencer run so no other methods need to be applied.

The rapid development of DNA sequencing techniques has allowed substantial improvement of the culture-independent identification of microbial pathogens. On the other hand, the advances in DNA sequencing techniques has allowed simultaneous investigation of millions of DNA fragments, enabling a rapid identification of all the microorganisms present in a given clinical sample. NGS-based techniques, especially NGS of the 16S rRNA gene, have been successfully applied to the comprehensive analysis of microbiomes not only from healthy people, but also from those associated with many diseases [48–50]. As sensitive NGS-based techniques enable accurate detection of the microbiome composition, it could lead to better understanding of the species content that might modulate growth, virulence, biofilm formation, quorum sensing, and antibiotic resistance [51]. In any case, identification of microbiome-constituents at the species or genera level is microbiologically not detailed enough. This is also because microbes are transmitted between hosts and have different virulence, fitness factors (e.g. tenacity), transmission power, and biological and epidemiological behavior.

## Conclusions

In conclusion, our study demonstrated a high reliability of NGS of the 16S–23S rRNA region sequencing in streptococci and enterococci identification at the species level. The method based on NGS of the 16S–23S rRNA region had undoubtedly one of the highest identification potential from all the methods used. We have developed a reference dataset of the 16S–23S rRNA region for 42 streptococcal and 9 enterococcal species, therefore, many clinically relevant streptococcal and enterococcal species can now be detected in a clinical sample. All diagnostic laboratories which have access to next generation sequencing will be able to introduce a highly precise, rapid and reliable method for identification of microorganisms and the obtained results will facilitate an unambiguous identification of many clinically significant streptococci and enterococci in all samples.

## Supplementary information

**Additional file 1. **Includes (i) matrixes with differences in the number of nucleotides and deletions between sequence pairs of all Sanger-based and NGS-based method, (ii) table with the length of 16S–23S rRNA region, 16S rRNA gene, intergenic spacer region and 23S rRNA region for all species, (iii) table with the intraspecies polymorphism of 16S–23S rRNA region sequence within *Streptococcus* and *Enterococcus* genera, (iv) the comparison between phylogenetic trees based on 16S–23S rRNA region and 16S rRNA, *rpoB*, *sodA* and *tuf* genes for both *Streptococcus* and *Enterococcus* species.

## Data Availability

The datasets generated for this study can be found in Genbank, MK330555-MK330596, MK322658-MK322666; MK330513-MK330554, MK322649-MK322657; MK322556-MK322597, MK308717-MK308725; MK322607-MK322648, MK322598-MK322606; MK322514-MK322555, MK308708-MK308716. The NGS data can be found in European Nucleotide Archive (ENA), PRJEB32803 (ERP115525).

## Abbreviations

ATCC: American Type Culture Collection
BLAST: Basic Local Alignment Search Tool
DSM: Leibniz Institute DSMZ-German Collection of Microorganisms and Cell Cultures (DSMZ)
GAS: Group A *Streptococcus*
GBS: Group B *Streptococcus*
BCCM (LMG): Belgian Coordinated Collection of Microorganisms
MALDI-TOF MS: Matrix-assisted laser desorption ionization–time of flight mass spectrometry
NGS: Next generation sequencing
