# Supplementary material for: Development of a reference data set for assigning Streptococcus and Enterococcus species based on next generation sequencing of the 16S–23S rRNA region
Source: Antimicrob Resist Infect Control. 2019 Nov 15;8:178. doi: 10.1186/s13756-019-0622-3 (PMC6858756; doi:10.1186/s13756-019-0622-3)

Additional file 1

Description for all matrixes: The part of the matrix from the upper right corner shows differences in the number of deletions between sequence pairs; the part of the matrix from the lower left corner shows differences in the number of nucleotides between the sequence pairs. The green color indicates species pairs that differ in the lowest number of nucleotides or deletions, the red color the pairs with the highest number of nucleotide or deletion differences between sequences.

Table S1

A matrix showing the nucleotides and deletions differences between pairs of 16S rRNA gene sequences for *Streptococcus* species.

		1	2	3	4	5	6	7	8	9	10	11	12	13	14	15	16	17	18	19	20	21	22	23	24	25	26	27	28	29	30	31	32	33	34	35	36	37	38	39	40	41	42
S._acidominimus	1		22	8	4	0	8	1	9	4	0	11	16	0	0	7	8	8	8	2	8	4	10	8	4	13	2	8	10	4	0	4	0	0	13	8	2	8	11	10	2	0	0
S._adjacens	2	211		18	22	22	18	23	17	22	22	17	10	22	22	25	26	18	26	22	18	22	16	24	22	19	22	26	16	22	22	22	22	22	11	26	22	18	19	28	22	22	22
S._anginosus	3	103	209		10	8	0	9	1	10	8	3	8	8	8	7	8	0	8	8	0	10	2	8	10	5	8	8	2	10	8	10	8	8	7	8	8	0	3	10	8	8	8
S._australis	4	88	194	103		4	10	5	11	0	4	13	18	4	4	9	6	10	6	2	10	0	12	10	0	13	2	6	12	0	4	0	4	4	15	6	2	10	13	8	2	4	4
S._canis	5	86	202	97	81		8	1	9	4	0	11	16	0	0	7	8	8	8	2	8	4	10	8	4	13	2	8	10	4	0	4	0	0	13	8	2	8	11	10	2	0	0
S._constellatus	6	88	194	62	74	95		9	1	10	8	3	8	8	8	7	8	0	8	8	0	10	2	8	10	5	8	8	2	10	8	10	8	8	7	8	8	0	3	10	8	8	8
S._cremoris	7	147	209	145	143	135	155		10	5	1	12	17	1	1	8	9	9	9	3	9	5	11	9	5	14	3	9	11	5	1	5	1	1	14	9	3	9	12	11	3	1	1
S._criceti	8	98	216	90	119	89	111	153		11	9	2	7	9	9	8	9	1	9	9	1	11	3	9	11	4	9	9	1	11	9	11	9	9	6	9	9	1	2	11	9	9	9
S._cristatus	9	78	197	82	40	83	81	136	96		4	13	18	4	4	9	6	10	6	2	10	0	12	10	0	13	2	6	12	0	4	0	4	4	15	6	2	10	13	8	2	4	4
S._difficilis	10	84	198	108	82	55	88	125	107	84		11	16	0	0	7	8	8	8	2	8	4	10	8	4	13	2	8	10	4	0	4	0	0	13	8	2	8	11	10	2	0	0
S._downei	11	117	210	97	112	101	93	161	86	101	109		7	11	11	10	11	3	11	11	3	13	5	11	13	6	11	11	3	13	11	13	11	11	6	11	11	3	2	13	11	11	11
S._durans	12	191	97	176	173	185	165	182	185	174	172	184		16	16	15	16	8	16	16	8	18	8	16	18	9	16	16	6	18	16	18	16	16	5	16	16	8	9	18	16	16	16
S._dysgalactiae	13	78	197	105	78	43	89	122	109	79	35	108	171		0	7	8	8	8	2	8	4	10	8	4	13	2	8	10	4	0	4	0	0	13	8	2	8	11	10	2	0	0
S._equi	14	104	228	88	104	62	108	134	86	91	76	116	195	78		7	8	8	8	2	8	4	10	8	4	13	2	8	10	4	0	4	0	0	13	8	2	8	11	10	2	0	0
S._equinus	15	83	207	89	79	91	85	139	103	66	91	99	180	90	95		7	7	7	7	7	9	9	7	9	12	7	7	9	9	7	9	7	7	14	7	7	7	10	9	7	7	7
S._gallolyticus	16	95	213	65	82	78	86	131	88	71	87	89	186	83	78	73		8	0	6	8	6	10	8	6	11	6	0	10	6	8	6	8	8	15	0	6	8	11	4	6	8	8
S._gordonii	17	88	197	62	53	83	59	153	92	48	91	91	167	89	101	80	74		8	8	0	10	2	8	10	5	8	8	2	10	8	10	8	8	7	8	8	0	3	10	8	8	8
S._infantarius	18	96	211	74	78	78	86	124	94	73	76	100	184	81	76	71	21	77		6	8	6	10	8	6	11	6	0	10	6	8	6	8	8	15	0	6	8	11	4	6	8	8
S._infantis	19	88	197	99	25	84	76	139	112	35	84	106	171	74	99	77	75	53	80		8	2	10	8	2	13	0	6	10	2	2	2	2	2	13	6	0	8	11	8	0	2	2
S._intermedius	20	90	203	48	76	82	23	154	101	74	96	88	173	93	99	89	81	44	80	81		10	2	8	10	5	8	8	2	10	8	10	8	8	7	8	8	0	3	10	8	8	8
S._mitis	21	94	193	93	21	87	71	144	115	42	82	107	171	77	96	81	79	54	81	27	77		12	10	0	13	2	6	12	0	4	0	4	4	15	6	2	10	13	8	2	4	4
S._mutans	22	122	217	96	112	119	107	152	104	98	119	99	194	121	107	106	99	84	93	107	102	108		8	12	5	10	10	2	12	10	12	10	10	7	10	10	2	5	12	10	10	10
S._oligofermentans	23	84	197	84	64	87	83	135	107	42	91	107	180	82	96	65	77	60	74	65	70	66	102		10	13	8	8	10	10	8	10	8	8	15	8	8	8	11	10	8	8	8
S._oralis	24	88	192	94	18	84	72	141	114	41	79	105	168	73	97	80	78	49	78	24	74	6	106	65		13	2	6	12	0	4	0	4	4	15	6	2	10	13	8	2	4	4
S._ovis	25	96	211	89	101	89	91	160	85	93	98	87	182	100	105	85	89	85	91	105	83	102	114	99	99		13	11	3	13	13	13	13	13	8	11	13	5	6	13	13	13	13
S._parasanguinis	26	70	188	87	50	78	76	131	99	49	76	105	160	68	90	70	73	64	73	48	78	48	110	62	43	96		6	10	2	2	2	2	2	13	6	0	8	11	8	0	2	2
S._pasteurianus	27	95	212	65	81	77	85	129	89	70	85	90	183	81	80	73	3	72	19	74	81	77	100	77	76	90	72		10	6	8	6	8	8	15	0	6	8	11	4	6	8	8
S._pluranimalium	28	112	197	99	118	93	104	159	96	104	107	91	181	110	106	96	98	96	92	120	96	115	114	99	112	75	99	99		12	10	12	10	10	5	10	10	2	3	12	10	10	10
S._pneumoniae	29	95	194	96	24	88	74	146	116	43	85	107	172	78	98	83	81	57	85	28	78	6	110	68	9	104	50	79	117		4	0	4	4	15	6	2	10	13	8	2	4	4
S._porcinus	30	78	206	105	79	45	94	141	111	88	49	122	179	48	61	82	86	93	77	89	98	78	124	92	75	99	74	85	101	81		4	0	0	13	8	2	8	11	10	2	0	0
S._pseudopneumoniae	31	92	191	93	21	87	69	144	116	41	82	105	170	77	96	79	77	53	81	24	72	5	108	65	5	99	47	77	113	6	78		4	4	15	6	2	10	13	8	2	4	4
S._pseudoporcinus	32	80	208	113	85	55	100	145	113	88	50	125	181	52	67	83	93	100	89	89	104	87	128	92	85	110	74	93	107	87	31	85		0	13	8	2	8	11	10	2	0	0
S._pyogenes	33	84	200	102	84	27	94	132	91	81	51	100	179	37	69	90	75	84	73	85	84	85	119	87	83	85	70	73	92	86	57	86	62		13	8	2	8	11	10	2	0	0
S._saccharolyticus	34	186	110	175	171	179	165	186	187	172	170	179	51	167	199	177	178	164	177	165	169	166	191	177	164	178	163	175	179	167	180	165	179	175		15	13	7	8	17	13	13	13
S._salivarius	35	93	205	81	68	83	95	140	93	61	101	107	177	96	88	83	59	73	54	72	79	70	100	73	66	77	65	57	88	72	83	70	98	80	176		6	8	11	4	6	8	8
S._sanguinis	36	76	195	86	32	67	62	140	98	36	78	99	174	71	86	70	67	46	72	25	65	34	96	61	29	93	38	66	105	34	73	31	74	74	169	66		8	11	8	0	2	2
S._sinensis	37	90	199	70	80	93	59	146	105	57	98	101	176	96	104	66	84	51	80	77	49	80	98	34	77	87	65	82	85	82	95	79	101	93	174	80	66		3	10	8	8	8
S._sobrinus	38	115	215	96	104	96	93	163	84	106	102	45	193	108	106	93	90	88	94	107	88	107	98	100	106	88	111	91	90	109	113	105	120	97	189	106	101	100		13	11	11	11
S._suis	39	71	198	92	77	81	83	143	91	68	72	107	171	80	92	57	71	78	61	81	83	83	118	71	78	84	59	69	94	86	74	82	75	76	169	68	69	79	102		8	10	10
S._tigurinus	40	87	195	96	24	81	75	138	110	34	81	105	171	71	96	80	74	52	79	3	78	24	104	64	21	104	47	73	118	25	86	21	86	82	166	71	22	78	106	81		2	2
S._uberis	41	81	212	99	77	38	99	141	100	84	65	110	182	57	61	85	74	86	75	87	84	85	117	89	81	87	73	76	96	88	37	83	37	44	182	74	73	97	104	71	84		0
S._urinalis	42	86	200	100	83	38	94	142	104	86	56	98	185	55	73	87	69	82	67	86	84	88	122	85	84	81	73	68	90	90	60	86	61	42	179	82	74	92	100	70	87	48	

Table S2

A matrix showing the nucleotides and deletions differences between pairs of 16S rRNA gene sequences for *Enterococcus* species.

		1	2	3	4	5	6	7	8	9
E._avium	1		0	0	3	3	3	3	3	0
E._casseliflavus	2	22		0	3	3	3	3	3	0
E._cecorum	3	59	53		3	3	3	3	3	0
E._durans	4	22	34	71		0	0	0	0	3
E._faecium	5	24	35	74	7		0	0	0	3
E._faecalis	6	66	62	90	59	55		0	0	3
E._hirae	7	20	34	71	5	7	56		0	3
E._porcinus	8	27	35	75	16	14	63	13		3
E._raffinosus	9	5	20	59	23	25	67	21	26	

Table S3

A matrix showing the nucleotides and deletions differences between pairs of *sodA* gene sequences for *Streptococcus* species.

		1	2	3	4	5	6	7	8	9	10	11	12	13	14	15	16	17	18	19	20	21	22	23	24	25	26	27	28	29	30	31	32	33	34	35	36	37	38	39	40	41	42
S._acidominimus	1		9	0	0	0	0	17	0	0	0	0	9	0	0	0	0	0	0	0	0	0	0	0	0	0	0	0	0	0	0	0	0	0	9	0	0	0	0	0	0	0	0
S._adjacens	2	162		9	9	9	9	14	9	9	9	9	0	9	9	9	9	9	9	9	9	9	9	9	9	9	9	9	9	9	9	9	9	9	0	9	9	9	9	9	9	9	9
S._anginosus	3	104	155		0	0	0	17	0	0	0	0	9	0	0	0	0	0	0	0	0	0	0	0	0	0	0	0	0	0	0	0	0	0	9	0	0	0	0	0	0	0	0
S._australis	4	103	141	96		0	0	17	0	0	0	0	9	0	0	0	0	0	0	0	0	0	0	0	0	0	0	0	0	0	0	0	0	0	9	0	0	0	0	0	0	0	0
S._canis	5	113	163	115	120		0	17	0	0	0	0	9	0	0	0	0	0	0	0	0	0	0	0	0	0	0	0	0	0	0	0	0	0	9	0	0	0	0	0	0	0	0
S._constellatus	6	105	152	53	93	122		17	0	0	0	0	9	0	0	0	0	0	0	0	0	0	0	0	0	0	0	0	0	0	0	0	0	0	9	0	0	0	0	0	0	0	0
S._cremoris	7	157	169	146	158	157	159		17	17	17	17	14	17	17	17	17	17	17	17	17	17	17	17	17	17	17	17	17	17	17	17	17	17	14	17	17	17	17	17	17	17	17
S._criceti	8	105	174	116	110	98	123	163		0	0	0	9	0	0	0	0	0	0	0	0	0	0	0	0	0	0	0	0	0	0	0	0	0	9	0	0	0	0	0	0	0	0
S._cristatus	9	93	151	91	52	117	86	158	109		0	0	9	0	0	0	0	0	0	0	0	0	0	0	0	0	0	0	0	0	0	0	0	0	9	0	0	0	0	0	0	0	0
S._difficilis	10	98	168	103	109	102	100	155	103	104		0	9	0	0	0	0	0	0	0	0	0	0	0	0	0	0	0	0	0	0	0	0	0	9	0	0	0	0	0	0	0	0
S._downei	11	110	172	115	114	101	121	164	62	112	103		9	0	0	0	0	0	0	0	0	0	0	0	0	0	0	0	0	0	0	0	0	0	9	0	0	0	0	0	0	0	0
S._durans	12	165	138	156	147	155	153	176	155	151	160	161		9	9	9	9	9	9	9	9	9	9	9	9	9	9	9	9	9	9	9	9	9	0	9	9	9	9	9	9	9	9
S._dysgalactiae	13	119	158	113	122	30	118	161	103	120	102	107	154		0	0	0	0	0	0	0	0	0	0	0	0	0	0	0	0	0	0	0	0	9	0	0	0	0	0	0	0	0
S._equi	14	124	166	122	123	71	130	165	114	120	115	107	160	78		0	0	0	0	0	0	0	0	0	0	0	0	0	0	0	0	0	0	0	9	0	0	0	0	0	0	0	0
S._equinus	15	85	172	117	120	114	115	167	101	114	91	97	167	115	129		0	0	0	0	0	0	0	0	0	0	0	0	0	0	0	0	0	0	9	0	0	0	0	0	0	0	0
S._gallolyticus	16	111	148	99	106	106	97	156	117	104	103	117	153	110	114	124		0	0	0	0	0	0	0	0	0	0	0	0	0	0	0	0	0	9	0	0	0	0	0	0	0	0
S._gordonii	17	98	138	79	71	115	72	149	105	67	93	118	146	119	129	102	103		0	0	0	0	0	0	0	0	0	0	0	0	0	0	0	0	9	0	0	0	0	0	0	0	0
S._infantarius	18	118	162	107	106	97	107	159	106	105	103	113	159	95	115	112	76	104		0	0	0	0	0	0	0	0	0	0	0	0	0	0	0	9	0	0	0	0	0	0	0	0
S._infantis	19	99	153	95	34	120	91	165	105	54	109	114	151	121	124	113	115	73	109		0	0	0	0	0	0	0	0	0	0	0	0	0	0	9	0	0	0	0	0	0	0	0
S._intermedius	20	107	152	64	96	123	38	151	120	88	104	130	158	125	130	122	102	70	108	89		0	0	0	0	0	0	0	0	0	0	0	0	0	9	0	0	0	0	0	0	0	0
S._mitis	21	97	145	90	29	116	84	156	106	49	105	115	145	117	126	106	101	66	103	24	83		0	0	0	0	0	0	0	0	0	0	0	0	9	0	0	0	0	0	0	0	0
S._mutans	22	101	168	111	113	82	110	155	96	110	93	100	165	88	106	97	93	107	97	115	111	107		0	0	0	0	0	0	0	0	0	0	0	9	0	0	0	0	0	0	0	0
S._oligofermentans	23	96	150	90	56	123	92	158	117	32	109	119	162	127	129	116	108	74	113	65	96	60	116		0	0	0	0	0	0	0	0	0	0	9	0	0	0	0	0	0	0	0
S._oralis	24	93	153	90	28	122	90	160	104	49	105	113	148	123	127	110	108	72	105	15	91	22	116	60		0	0	0	0	0	0	0	0	0	9	0	0	0	0	0	0	0	0
S._ovis	25	85	157	93	90	111	101	159	105	88	90	111	162	108	113	90	106	87	107	85	103	84	107	90	88		0	0	0	0	0	0	0	0	9	0	0	0	0	0	0	0	0
S._parasanguinis	26	99	151	91	49	111	81	150	102	54	95	114	148	117	122	107	106	64	94	53	83	43	106	63	48	95		0	0	0	0	0	0	0	9	0	0	0	0	0	0	0	0
S._pasteurianus	27	108	154	104	111	107	98	164	113	105	103	113	149	110	117	120	16	102	74	113	107	102	96	109	111	110	107		0	0	0	0	0	0	9	0	0	0	0	0	0	0	0
S._pluranimalium	28	112	150	111	104	97	121	153	105	106	96	96	148	92	109	111	113	114	108	108	125	104	102	115	111	101	105	116		0	0	0	0	0	9	0	0	0	0	0	0	0	0
S._pneumoniae	29	92	145	92	34	113	88	154	105	47	105	111	150	115	124	103	106	66	106	25	90	13	109	60	26	84	48	107	98		0	0	0	0	9	0	0	0	0	0	0	0	0
S._porcinus	30	112	160	113	123	93	105	142	110	111	100	111	162	92	110	109	107	110	114	121	107	111	94	120	119	110	117	110	106	117		0	0	0	9	0	0	0	0	0	0	0	0
S._pseudopneumoniae	31	93	147	93	36	114	89	155	106	49	106	112	152	117	125	104	108	67	108	27	91	15	110	62	28	86	49	109	99	2	118		0	0	9	0	0	0	0	0	0	0	0
S._pseudoporcinus	32	109	166	117	118	90	109	152	104	108	101	112	170	88	102	107	111	113	110	116	111	113	93	117	119	104	116	114	112	118	27	119		0	9	0	0	0	0	0	0	0	0
S._pyogenes	33	118	159	114	123	31	119	162	102	121	103	106	155	1	79	114	111	120	96	122	126	118	87	128	124	109	118	111	93	114	93	116	88		9	0	0	0	0	0	0	0	0
S._saccharolyticus	34	149	125	149	147	155	152	162	147	143	146	161	98	156	165	153	161	137	158	145	144	142	158	146	142	157	136	161	145	144	158	145	162	157		9	9	9	9	9	9	9	9
S._salivarius	35	97	151	105	86	96	100	158	95	96	93	99	156	94	109	106	88	89	88	92	105	84	82	102	90	84	101	92	96	88	102	90	97	95	162		0	0	0	0	0	0	0
S._sanguinis	36	104	149	90	82	122	77	156	106	80	93	113	150	123	125	118	103	57	117	83	85	77	103	80	82	104	71	106	115	82	121	83	121	124	150	103		0	0	0	0	0	0
S._sinensis	37	104	163	80	84	125	79	160	117	67	106	116	164	130	122	119	106	70	109	84	82	80	111	54	85	96	77	109	118	86	115	87	112	131	156	98	78		0	0	0	0	0
S._sobrinus	38	101	172	112	112	103	120	173	64	104	93	59	155	103	114	94	115	106	115	107	130	104	108	116	106	105	105	108	92	102	109	103	105	104	159	95	111	115		0	0	0	0
S._suis	39	67	164	103	96	118	104	154	94	86	92	86	159	119	116	74	100	91	106	98	104	89	95	88	94	71	92	100	98	90	106	92	108	120	152	90	93	97	90		0	0	0
S._tigurinus	40	96	146	95	34	122	86	161	109	50	113	117	147	122	128	112	105	74	107	22	90	21	116	65	17	93	55	109	109	25	119	27	121	123	146	92	82	85	111	98		0	0
S._uberis	41	113	160	112	111	85	113	149	100	112	92	101	157	87	104	103	111	105	109	111	112	102	93	121	112	98	103	111	89	103	86	105	93	86	142	97	114	117	109	103	108		0
S._urinalis	42	98	167	117	115	98	108	154	113	109	98	113	149	101	120	93	104	105	96	119	108	108	96	119	111	99	100	106	106	113	98	114	102	102	151	102	110	119	113	89	113	99	

Table S4

A matrix showing the nucleotides and deletions differences between pairs of *sodA* gene sequences for *Enterococcus* species.

		1	2	3	4	5	6	7	8	9
E._avium	1		0	0	0	0	0	0	0	0
E._casseliflavus	2	96		0	0	0	0	0	0	0
E._cecorum	3	119	114		0	0	0	0	0	0
E._durans	4	112	98	121		0	0	0	0	0
E._faecium	5	99	77	106	95		0	0	0	0
E._faecalis	6	97	75	106	94	5		0	0	0
E._hirae	7	96	80	105	62	78	80		0	0
E._porcinus	8	118	105	113	70	99	100	60		0
E._raffinosus	9	52	84	124	100	94	93	90	114	

Table S5

A matrix showing the nucleotides and deletions differences between pairs of *tuf* gene sequences for *Streptococcus* species.

		1	2	3	4	5	6	7	8	9	10	11	12	13	14	15	16	17	18	19	20	21	22	23	24	25	26	27	28	29	30	31	32	33	34	35	36	37	38	39	40	41	42
S._acidominimus	1		0	0	0	0	0	0	0	0	0	0	0	0	0	0	0	0	0	0	0	0	0	0	0	0	0	0	0	0	0	0	0	0	0	0	0	0	0	0	0	0	0
S._adjacens	2	178		0	0	0	0	0	0	0	0	0	0	0	0	0	0	0	0	0	0	0	0	0	0	0	0	0	0	0	0	0	0	0	0	0	0	0	0	0	0	0	0
S._anginosus	3	74	178		0	0	0	0	0	0	0	0	0	0	0	0	0	0	0	0	0	0	0	0	0	0	0	0	0	0	0	0	0	0	0	0	0	0	0	0	0	0	0
S._australis	4	55	189	62		0	0	0	0	0	0	0	0	0	0	0	0	0	0	0	0	0	0	0	0	0	0	0	0	0	0	0	0	0	0	0	0	0	0	0	0	0	0
S._canis	5	73	182	79	61		0	0	0	0	0	0	0	0	0	0	0	0	0	0	0	0	0	0	0	0	0	0	0	0	0	0	0	0	0	0	0	0	0	0	0	0	0
S._constellatus	6	76	177	10	62	81		0	0	0	0	0	0	0	0	0	0	0	0	0	0	0	0	0	0	0	0	0	0	0	0	0	0	0	0	0	0	0	0	0	0	0	0
S._cremoris	7	145	190	142	140	147	140		0	0	0	0	0	0	0	0	0	0	0	0	0	0	0	0	0	0	0	0	0	0	0	0	0	0	0	0	0	0	0	0	0	0	0
S._criceti	8	77	195	82	82	99	82	154		0	0	0	0	0	0	0	0	0	0	0	0	0	0	0	0	0	0	0	0	0	0	0	0	0	0	0	0	0	0	0	0	0	0
S._cristatus	9	55	185	72	43	63	68	137	79		0	0	0	0	0	0	0	0	0	0	0	0	0	0	0	0	0	0	0	0	0	0	0	0	0	0	0	0	0	0	0	0	0
S._difficilis	10	64	178	67	59	57	66	148	85	52		0	0	0	0	0	0	0	0	0	0	0	0	0	0	0	0	0	0	0	0	0	0	0	0	0	0	0	0	0	0	0	0
S._downei	11	83	199	78	88	106	82	160	25	80	80		0	0	0	0	0	0	0	0	0	0	0	0	0	0	0	0	0	0	0	0	0	0	0	0	0	0	0	0	0	0	0
S._durans	12	176	103	186	181	186	187	187	199	183	176	204		0	0	0	0	0	0	0	0	0	0	0	0	0	0	0	0	0	0	0	0	0	0	0	0	0	0	0	0	0	0
S._dysgalactiae	13	65	186	81	56	34	82	142	101	61	48	105	179		0	0	0	0	0	0	0	0	0	0	0	0	0	0	0	0	0	0	0	0	0	0	0	0	0	0	0	0	0
S._equi	14	78	193	82	65	61	84	155	99	75	64	100	192	65		0	0	0	0	0	0	0	0	0	0	0	0	0	0	0	0	0	0	0	0	0	0	0	0	0	0	0	0
S._equinus	15	53	175	89	55	75	89	142	86	58	67	87	183	74	84		0	0	0	0	0	0	0	0	0	0	0	0	0	0	0	0	0	0	0	0	0	0	0	0	0	0	0
S._gallolyticus	16	66	183	53	55	69	55	134	70	52	51	73	185	66	67	67		0	0	0	0	0	0	0	0	0	0	0	0	0	0	0	0	0	0	0	0	0	0	0	0	0	0
S._gordonii	17	54	176	65	37	64	67	131	81	34	58	84	179	61	72	61	48		0	0	0	0	0	0	0	0	0	0	0	0	0	0	0	0	0	0	0	0	0	0	0	0	0
S._infantarius	18	73	186	62	62	69	62	133	75	57	58	82	191	72	74	65	18	59		0	0	0	0	0	0	0	0	0	0	0	0	0	0	0	0	0	0	0	0	0	0	0	0
S._infantis	19	59	185	64	35	62	66	136	84	42	61	88	178	57	66	65	56	47	65		0	0	0	0	0	0	0	0	0	0	0	0	0	0	0	0	0	0	0	0	0	0	0
S._intermedius	20	71	182	17	63	84	19	142	84	69	67	84	183	81	84	90	54	66	61	64		0	0	0	0	0	0	0	0	0	0	0	0	0	0	0	0	0	0	0	0	0	0
S._mitis	21	61	186	64	24	65	66	134	88	45	63	93	176	58	75	60	58	37	65	30	63		0	0	0	0	0	0	0	0	0	0	0	0	0	0	0	0	0	0	0	0	0
S._mutans	22	89	175	79	98	97	80	160	81	88	87	83	185	104	97	98	67	90	73	97	80	101		0	0	0	0	0	0	0	0	0	0	0	0	0	0	0	0	0	0	0	0
S._oligofermentans	23	52	185	70	39	61	72	137	78	14	53	83	179	58	72	58	53	32	57	41	71	44	91		0	0	0	0	0	0	0	0	0	0	0	0	0	0	0	0	0	0	0
S._oralis	24	55	184	64	34	58	66	135	92	38	58	91	177	51	72	59	60	41	67	18	63	24	105	35		0	0	0	0	0	0	0	0	0	0	0	0	0	0	0	0	0	0
S._ovis	25	45	182	79	69	72	81	132	81	57	71	86	185	70	87	59	69	46	77	63	81	60	92	56	59		0	0	0	0	0	0	0	0	0	0	0	0	0	0	0	0	0
S._parasanguinis	26	50	176	63	39	60	65	145	76	44	54	77	179	57	66	57	50	38	56	45	66	49	89	38	44	60		0	0	0	0	0	0	0	0	0	0	0	0	0	0	0	0
S._pasteurianus	27	66	185	56	57	71	58	139	70	51	51	71	185	67	73	66	10	55	23	58	56	63	68	53	61	76	54		0	0	0	0	0	0	0	0	0	0	0	0	0	0	0
S._pluranimalium	28	66	177	78	80	84	78	136	75	80	67	80	182	80	84	69	66	70	70	81	79	79	90	79	83	70	72	71		0	0	0	0	0	0	0	0	0	0	0	0	0	0
S._pneumoniae	29	61	187	66	28	63	68	133	93	47	64	96	175	57	75	64	58	38	65	32	61	11	103	44	21	60	49	63	80		0	0	0	0	0	0	0	0	0	0	0	0	0
S._porcinus	30	63	184	77	59	60	79	148	93	64	51	92	184	47	71	76	70	68	76	58	79	66	91	62	55	71	63	68	74	64		0	0	0	0	0	0	0	0	0	0	0	0
S._pseudopneumoniae	31	60	188	66	25	65	68	135	90	46	64	95	177	56	75	61	59	37	66	29	65	7	102	43	21	59	48	64	79	6	61		0	0	0	0	0	0	0	0	0	0	0
S._pseudoporcinus	32	62	183	78	62	53	79	147	93	64	47	91	180	39	70	75	71	68	79	58	76	60	95	63	51	68	58	71	73	59	24	60		0	0	0	0	0	0	0	0	0	0
S._pyogenes	33	69	185	79	61	19	83	145	99	63	55	105	181	21	55	75	65	62	68	60	83	61	103	59	56	72	58	70	80	62	54	61	47		0	0	0	0	0	0	0	0	0
S._saccharolyticus	34	183	91	187	195	193	188	194	192	187	187	198	77	189	205	184	190	191	188	188	184	191	180	186	187	190	185	187	176	189	187	190	182	192		0	0	0	0	0	0	0	0
S._salivarius	35	68	178	67	52	69	65	145	69	56	56	76	181	67	74	56	61	56	66	61	69	61	91	55	56	68	48	65	72	62	69	60	68	69	183		0	0	0	0	0	0	0
S._sanguinis	36	57	183	72	47	68	72	137	82	36	67	87	178	65	83	70	60	22	67	53	73	44	95	34	49	51	42	63	77	44	70	43	73	64	189	65		0	0	0	0	0	0
S._sinensis	37	68	184	75	59	84	72	140	81	43	66	79	186	78	76	75	59	46	63	57	72	56	80	50	56	70	48	59	84	60	75	57	79	80	191	68	50		0	0	0	0	0
S._sobrinus	38	81	196	84	88	101	88	153	31	82	87	38	195	101	98	86	75	85	77	90	86	93	80	83	96	86	81	75	77	95	96	95	94	99	193	70	82	83		0	0	0	0
S._suis	39	44	178	83	52	73	83	142	84	55	66	85	184	68	76	16	64	55	65	63	84	57	95	53	56	53	50	65	65	56	74	54	71	71	185	53	64	72	83		0	0	0
S._tigurinus	40	58	184	63	34	61	65	135	83	41	62	89	177	56	65	66	55	46	64	1	63	29	96	40	19	62	44	57	80	31	59	28	59	59	187	60	52	56	89	62		0	0
S._uberis	41	61	177	72	57	40	73	146	93	54	54	94	184	41	73	65	65	63	66	54	71	59	100	52	50	70	51	61	75	57	50	58	41	45	185	61	68	71	97	63	53		0
S._urinalis	42	74	182	67	77	76	68	138	80	74	54	85	183	68	81	88	55	69	67	78	67	78	86	74	80	86	71	55	72	77	72	81	67	72	182	75	78	75	92	84	77	59	

Table S6

A matrix showing the nucleotides and deletions differences between pairs of *tuf* gene sequences for *Enterococcus* species.

		1	2	3	4	5	6	7	8	9
E._avium	1		0	0	0	0	0	0	0	0
E._casseliflavus	2	70		0	0	0	0	0	0	0
E._cecorum	3	71	76		0	0	0	0	0	0
E._durans	4	80	75	72		0	0	0	0	0
E._faecium	5	86	71	75	28		0	0	0	0
E._faecalis	6	69	73	75	89	93		0	0	0
E._hirae	7	80	73	70	10	26	87		0	0
E._porcinus	8	107	99	99	57	72	114	57		0
E._raffinosus	9	19	73	72	81	83	74	82	104	

Table S7

A matrix showing the nucleotides and deletions differences between pairs of *rpoB* gene sequences for *Streptococcus* species.

		1	2	3	4	5	6	7	8	9	10	11	12	13	14	15	16	17	18	19	20	21	22	23	24	25	26	27	28	29	30	31	32	33	34	35	36	37	38	39	40	41	42
S._acidominimus	1		0	0	0	0	0	0	0	0	0	0	0	0	0	0	0	0	0	0	0	0	0	0	0	0	0	0	0	0	0	0	0	0	0	0	0	0	0	0	0	0	0
S._adjacens	2	172		0	0	0	0	0	0	0	0	0	0	0	0	0	0	0	0	0	0	0	0	0	0	0	0	0	0	0	0	0	0	0	0	0	0	0	0	0	0	0	0
S._anginosus	3	113	172		0	0	0	0	0	0	0	0	0	0	0	0	0	0	0	0	0	0	0	0	0	0	0	0	0	0	0	0	0	0	0	0	0	0	0	0	0	0	0
S._australis	4	91	165	103		0	0	0	0	0	0	0	0	0	0	0	0	0	0	0	0	0	0	0	0	0	0	0	0	0	0	0	0	0	0	0	0	0	0	0	0	0	0
S._canis	5	109	160	108	99		0	0	0	0	0	0	0	0	0	0	0	0	0	0	0	0	0	0	0	0	0	0	0	0	0	0	0	0	0	0	0	0	0	0	0	0	0
S._constellatus	6	110	175	58	109	112		0	0	0	0	0	0	0	0	0	0	0	0	0	0	0	0	0	0	0	0	0	0	0	0	0	0	0	0	0	0	0	0	0	0	0	0
S._cremoris	7	134	140	148	133	132	153		0	0	0	0	0	0	0	0	0	0	0	0	0	0	0	0	0	0	0	0	0	0	0	0	0	0	0	0	0	0	0	0	0	0	0
S._criceti	8	113	178	132	122	114	140	156		0	0	0	0	0	0	0	0	0	0	0	0	0	0	0	0	0	0	0	0	0	0	0	0	0	0	0	0	0	0	0	0	0	0
S._cristatus	9	97	175	104	78	91	106	136	112		0	0	0	0	0	0	0	0	0	0	0	0	0	0	0	0	0	0	0	0	0	0	0	0	0	0	0	0	0	0	0	0	0
S._difficilis	10	113	165	105	100	84	113	127	123	119		0	0	0	0	0	0	0	0	0	0	0	0	0	0	0	0	0	0	0	0	0	0	0	0	0	0	0	0	0	0	0	0
S._downei	11	117	180	138	129	111	139	167	70	115	130		0	0	0	0	0	0	0	0	0	0	0	0	0	0	0	0	0	0	0	0	0	0	0	0	0	0	0	0	0	0	0
S._durans	12	153	123	149	148	140	149	139	154	141	142	163		0	0	0	0	0	0	0	0	0	0	0	0	0	0	0	0	0	0	0	0	0	0	0	0	0	0	0	0	0	0
S._dysgalactiae	13	107	167	93	86	47	103	136	108	94	96	107	144		0	0	0	0	0	0	0	0	0	0	0	0	0	0	0	0	0	0	0	0	0	0	0	0	0	0	0	0	0
S._equi	14	121	180	120	119	95	117	154	112	108	104	118	154	90		0	0	0	0	0	0	0	0	0	0	0	0	0	0	0	0	0	0	0	0	0	0	0	0	0	0	0	0
S._equinus	15	103	162	111	96	87	111	148	129	97	103	129	157	97	115		0	0	0	0	0	0	0	0	0	0	0	0	0	0	0	0	0	0	0	0	0	0	0	0	0	0	0
S._gallolyticus	16	108	161	88	76	82	94	127	121	84	99	123	139	75	104	104		0	0	0	0	0	0	0	0	0	0	0	0	0	0	0	0	0	0	0	0	0	0	0	0	0	0
S._gordonii	17	100	172	110	88	100	113	141	111	69	109	117	154	91	103	96	97		0	0	0	0	0	0	0	0	0	0	0	0	0	0	0	0	0	0	0	0	0	0	0	0	0
S._infantarius	18	102	158	96	72	77	106	122	116	90	98	118	139	79	113	97	46	108		0	0	0	0	0	0	0	0	0	0	0	0	0	0	0	0	0	0	0	0	0	0	0	0
S._infantis	19	101	170	111	51	103	113	135	123	71	110	118	151	94	115	94	90	91	82		0	0	0	0	0	0	0	0	0	0	0	0	0	0	0	0	0	0	0	0	0	0	0
S._intermedius	20	116	179	77	118	127	81	151	135	112	116	147	155	121	131	122	102	120	104	122		0	0	0	0	0	0	0	0	0	0	0	0	0	0	0	0	0	0	0	0	0	0
S._mitis	21	89	170	103	48	98	101	128	118	70	100	120	141	89	110	93	80	87	80	37	122		0	0	0	0	0	0	0	0	0	0	0	0	0	0	0	0	0	0	0	0	0
S._mutans	22	116	167	114	112	101	121	145	118	115	98	123	154	92	92	101	109	111	105	113	127	113		0	0	0	0	0	0	0	0	0	0	0	0	0	0	0	0	0	0	0	0
S._oligofermentans	23	102	178	109	71	91	107	140	126	53	119	122	150	84	110	99	90	66	96	72	124	65	120		0	0	0	0	0	0	0	0	0	0	0	0	0	0	0	0	0	0	0
S._oralis	24	102	166	112	47	94	102	135	121	76	110	122	148	86	107	99	84	83	80	33	119	43	109	63		0	0	0	0	0	0	0	0	0	0	0	0	0	0	0	0	0	0
S._ovis	25	99	181	119	92	107	111	151	114	93	106	119	164	106	110	94	102	98	106	97	120	96	118	100	97		0	0	0	0	0	0	0	0	0	0	0	0	0	0	0	0	0
S._parasanguinis	26	101	177	96	90	96	92	138	128	82	105	130	142	96	112	97	82	82	85	92	99	86	128	92	84	90		0	0	0	0	0	0	0	0	0	0	0	0	0	0	0	0
S._pasteurianus	27	109	162	87	77	83	93	125	123	85	96	122	140	76	106	103	3	98	43	89	99	81	108	91	85	104	79		0	0	0	0	0	0	0	0	0	0	0	0	0	0	0
S._pluranimalium	28	102	161	108	88	73	115	130	115	103	91	123	142	75	91	95	84	109	82	103	114	97	90	106	97	104	98	84		0	0	0	0	0	0	0	0	0	0	0	0	0	0
S._pneumoniae	29	92	169	110	50	97	107	128	121	74	104	120	143	88	114	91	83	93	77	39	123	32	110	69	39	100	92	82	90		0	0	0	0	0	0	0	0	0	0	0	0	0
S._porcinus	30	108	157	124	97	97	129	124	118	111	108	124	142	104	116	117	87	112	88	112	126	106	108	113	102	113	108	89	100	104		0	0	0	0	0	0	0	0	0	0	0	0
S._pseudopneumoniae	31	91	160	107	43	100	103	129	124	75	101	126	139	90	114	98	78	92	78	36	124	26	106	69	37	97	88	79	96	37	105		0	0	0	0	0	0	0	0	0	0	0
S._pseudoporcinus	32	100	158	117	101	93	122	120	119	99	96	117	148	97	114	107	93	105	102	106	121	106	104	104	102	105	108	94	96	98	76	106		0	0	0	0	0	0	0	0	0	0
S._pyogenes	33	120	174	97	96	63	103	146	110	98	103	112	147	39	88	106	75	103	84	104	121	98	100	98	95	108	104	77	83	97	102	103	101		0	0	0	0	0	0	0	0	0
S._saccharolyticus	34	158	121	165	165	144	173	153	169	160	159	186	98	150	165	165	148	167	146	168	173	171	167	168	166	181	164	150	151	164	143	170	150	157		0	0	0	0	0	0	0	0
S._salivarius	35	93	154	92	67	73	92	131	112	76	85	112	140	71	91	88	55	90	59	75	102	70	107	81	64	90	67	56	66	69	93	72	94	72	151		0	0	0	0	0	0	0
S._sanguinis	36	96	182	110	95	108	114	140	117	74	111	120	160	106	118	98	100	61	109	99	112	101	123	72	97	91	91	101	114	96	117	106	98	113	170	92		0	0	0	0	0	0
S._sinensis	37	98	177	113	92	107	111	143	115	70	115	120	147	103	120	97	102	61	112	89	117	88	127	66	93	85	86	105	124	98	114	99	100	104	170	94	47		0	0	0	0	0
S._sobrinus	38	112	182	139	116	112	135	150	73	100	124	66	163	114	109	125	118	113	117	116	142	115	119	112	113	111	116	120	114	117	115	118	110	117	167	104	114	115		0	0	0	0
S._suis	39	107	163	114	95	82	111	152	128	94	100	123	150	90	114	17	104	96	96	87	117	94	106	100	92	92	92	103	90	86	120	97	104	101	159	80	94	95	121		0	0	0
S._tigurinus	40	100	166	114	46	89	106	132	121	74	101	118	146	80	114	98	89	84	83	30	124	43	111	64	37	92	93	88	95	43	113	38	102	94	168	73	89	85	114	89		0	0
S._uberis	41	115	149	128	113	115	121	131	123	119	110	140	147	117	115	121	104	113	113	113	122	110	112	115	106	123	119	104	103	111	96	110	97	119	149	107	119	122	131	124	111		0
S._urinalis	42	110	145	107	106	98	116	132	118	110	91	126	132	97	111	110	91	117	92	111	117	104	110	112	107	111	109	92	96	110	94	108	98	101	146	93	120	116	116	115	109	95	

Table S8

A matrix showing the nucleotides and deletions differences between pairs of *rpoB* gene sequences for *Enterococcus* species.

		1	2	3	4	5	6	7	8	9
E._avium	1		0	0	0	0	0	0	0	0
E._casseliflavus	2	118		0	0	0	0	0	0	0
E._cecorum	3	113	110		0	0	0	0	0	0
E._durans	4	108	98	98		0	0	0	0	0
E._faecium	5	114	99	101	62		0	0	0	0
E._faecalis	6	103	94	94	99	92		0	0	0
E._hirae	7	116	115	94	69	71	104		0	0
E._porcinus	8	114	113	94	83	77	83	71		0
E._raffinosus	9	64	102	113	103	118	92	117	109	

Table S9

A matrix showing the nucleotides and deletions differences between pairs of 16S-23S rRNA region sequences for *Streptococcus* species.

		1	2	3	4	5	6	7	8	9	10	11	12	13	14	15	16	17	18	19	20	21
S._acidominimus	1		425	111	244	279	170	287	235	236	287	236	322	279	167	258	262	247	270	240	217	240
S._adjacens	2	1065		372	241	274	301	266	554	259	274	479	143	274	384	547	215	244	219	239	256	237
S._anginosus	3	415	991		167	258	79	234	292	159	268	301	267	258	86	343	203	152	205	163	128	169
S._australis	4	495	830	391		157	94	95	435	30	159	352	166	157	223	492	58	35	60	4	51	10
S._canis	5	570	870	519	392		209	202	414	165	14	363	187	2	264	451	139	158	129	153	180	159
S._constellatus	6	456	905	248	292	474		161	355	90	213	288	202	209	155	406	128	81	132	90	53	96
S._cremoris	7	814	951	740	588	682	666		434	101	204	355	177	204	282	439	123	110	125	91	122	93
S._criceti	8	717	1209	714	784	782	753	983		441	418	115	421	412	258	251	403	428	403	433	408	439
S._cristatus	9	456	841	349	178	407	272	592	782		169	350	182	165	219	486	72	23	72	26	47	32
S._difficilis	10	574	883	549	382	211	482	683	793	404		367	189	14	268	457	145	164	135	155	182	161
S._downei	11	694	1124	678	662	719	640	915	419	656	710		356	361	281	266	328	341	328	350	325	356
S._durans	12	923	573	848	680	751	775	797	1067	712	738	985		187	279	430	138	165	136	162	177	162
S._dysgalactiae	13	563	881	538	381	101	482	660	798	408	175	729	743		266	451	141	160	131	153	180	159
S._equi	14	556	1000	423	500	493	501	770	697	504	521	692	860	520		355	199	228	195	221	206	225
S._equinus	15	595	1194	643	714	728	712	968	817	712	740	781	1047	721	723		462	481	462	488	453	486
S._gallolyticus	16	542	851	432	293	361	395	601	745	305	363	651	700	371	439	711		71	10	56	85	62
S._gordonii	17	478	834	331	174	402	273	625	764	132	394	636	689	403	495	725	298		71	31	36	37
S._infantarius	18	553	838	445	281	352	399	589	748	299	336	659	698	358	437	706	61	301		58	89	64
S._infantis	19	490	832	381	45	388	289	584	781	171	383	656	680	377	491	717	285	172	284		47	8
S._intermedius	20	477	866	270	239	427	157	626	770	224	444	645	732	448	511	733	333	195	335	240		49
S._mitis	21	501	827	386	52	397	294	584	779	182	385	660	678	383	490	712	294	186	291	58	248	
S._mutans	22	665	1002	510	548	638	537	823	740	544	637	709	892	645	514	847	531	523	535	542	540	555
S._oligofermentans	23	464	844	357	190	405	285	589	797	49	405	669	718	404	507	710	314	151	305	192	226	202
S._oralis	24	492	823	388	48	384	293	587	783	178	380	655	678	370	487	705	292	179	285	53	239	27
S._ovis	25	567	877	520	373	352	455	708	739	386	374	647	761	356	549	651	388	374	388	374	415	379
S._parasanguinis	26	486	833	381	156	384	299	574	754	210	385	654	680	379	477	715	268	222	270	154	234	166
S._pasteurianus	27	543	844	431	285	359	393	597	751	300	358	659	689	367	442	708	28	297	59	280	331	289
S._pluranimalium	28	585	891	541	432	271	493	706	764	416	275	688	759	281	545	747	377	406	363	431	449	427
S._pneumoniae	29	502	830	386	63	394	299	589	784	188	387	661	681	387	486	717	296	189	297	62	249	22
S._porcinus	30	580	1088	551	622	428	599	847	760	610	502	728	945	455	541	633	593	614	577	624	626	614
S._pseudopneumoniae	31	503	828	385	57	394	291	587	780	183	384	657	683	383	488	717	295	187	295	56	248	18
S._pseudoporcinus	32	539	1056	513	572	384	568	806	769	561	431	751	898	393	504	652	547	567	540	571	591	566
S._pyogenes	33	525	1008	486	509	265	525	804	735	522	317	709	876	254	457	696	478	513	467	513	517	520
S._saccharolyticus	34	989	500	909	734	795	835	870	1144	761	780	1055	274	786	936	1123	739	743	731	733	787	733
S._salivarius	35	533	829	451	243	362	399	594	726	280	347	649	676	365	451	684	169	279	156	249	321	256
S._sanguinis	36	467	856	362	191	408	260	596	784	133	415	663	714	415	511	730	312	153	315	180	199	197
S._sinensis	37	474	853	346	219	413	264	605	800	89	417	661	725	426	519	714	327	149	317	213	207	228
S._sobrinus	38	672	1022	648	549	560	588	818	534	574	546	317	877	571	659	783	553	534	551	552	567	556
S._suis	39	635	945	641	478	453	580	792	886	476	449	820	837	432	669	429	475	487	459	484	535	472
S._tigurinus	40	495	830	384	45	385	291	581	780	167	378	653	680	370	489	717	280	170	278	13	236	56
S._uberis	41	594	943	542	439	236	524	721	784	455	308	712	806	270	542	721	394	442	391	444	475	440
S._urinalis	42	598	875	541	409	188	481	684	781	422	187	705	758	189	519	739	352	402	344	407	426	411

		22	23	24	25	26	27	28	29	30	31	32	33	34	35	36	37	38	39	40	41	42
S._acidominimus	1	182	235	238	283	237	262	290	240	182	242	168	150	405	269	223	242	250	390	241	274	290
S._adjacens	2	359	256	237	298	244	213	275	237	455	237	417	399	42	216	248	251	371	343	238	329	273
S._anginosus	3	101	158	165	260	162	201	261	167	185	165	153	119	346	204	166	149	319	385	164	253	267
S._australis	4	202	29	6	163	13	56	164	10	332	8	294	262	219	55	39	38	262	280	5	204	164
S._canis	5	257	160	155	108	158	135	39	157	265	155	203	147	242	136	160	161	227	195	152	65	17
S._constellatus	6	132	91	92	205	89	126	210	96	252	94	222	190	275	129	101	82	260	326	91	238	216
S._cremoris	7	263	100	93	220	94	121	213	95	293	93	259	303	244	126	92	109	269	303	92	207	211
S._criceti	8	263	442	437	400	426	407	399	439	183	437	239	277	524	400	430	433	241	525	432	363	421
S._cristatus	9	200	5	28	161	29	70	176	32	324	30	292	260	235	69	23	12	272	278	25	204	172
S._difficilis	10	263	164	157	104	160	141	33	159	271	157	209	153	242	138	162	165	231	197	154	71	13
S._downei	11	270	351	354	327	343	332	348	356	204	354	266	300	449	331	341	342	146	458	349	320	370
S._durans	12	258	179	162	219	167	136	182	162	334	162	298	298	115	135	171	172	250	284	161	234	192
S._dysgalactiae	13	257	160	155	108	156	137	39	157	267	155	205	145	242	136	160	161	225	195	152	67	15
S._equi	14	83	220	221	272	218	197	257	225	189	225	159	123	360	206	228	227	331	397	220	261	267
S._equinus	15	378	483	486	457	485	462	452	486	198	488	258	318	531	459	473	476	328	324	487	404	464
S._gallolyticus	16	184	73	58	149	53	4	146	62	306	62	268	246	195	19	75	80	238	258	55	176	148
S._gordonii	17	191	24	33	156	34	71	161	37	325	35	293	265	218	72	38	15	253	279	30	199	167
S._infantarius	18	192	71	60	141	55	10	138	64	304	64	266	238	191	17	77	78	236	250	57	170	138
S._infantis	19	198	25	4	161	11	54	162	8	328	6	290	258	215	55	35	34	260	276	1	200	160
S._intermedius	20	175	46	45	164	44	83	183	47	301	49	271	241	230	84	54	37	267	285	48	217	185
S._mitis	21	206	31	4	165	17	60	168	2	334	4	296	264	215	61	41	40	262	272	9	206	166
S._mutans	22		201	202	265	197	188	248	206	220	204	190	164	335	195	209	194	292	394	197	270	262
S._oligofermentans	23	554		27	160	30	69	175	29	319	27	287	255	232	68	22	9	273	277	24	199	167
S._oralis	24	553	197		163	15	56	164	4	330	6	292	260	215	57	37	36	260	274	5	202	162
S._ovis	25	642	393	378		154	147	97	163	315	161	257	201	264	142	150	151	229	161	160	143	113
S._parasanguinis	26	557	221	166	381		51	165	19	329	17	291	257	220	52	34	37	257	271	12	201	163
S._pasteurianus	27	537	312	285	391	267		144	60	304	60	266	242	193	15	73	76	242	256	53	178	144
S._pluranimalium	28	634	410	422	353	408	378		168	294	166	234	176	241	141	171	168	212	204	161	100	40
S._pneumoniae	29	558	207	35	381	179	291	433		332	2	294	262	215	61	39	38	262	272	9	204	164
S._porcinus	30	711	612	605	610	612	587	554	613		330	64	130	425	307	319	320	264	408	327	210	274
S._pseudopneumoniae	31	556	202	33	375	167	291	429	20	610		292	260	215	61	37	36	264	274	7	202	162
S._pseudoporcinus	32	670	559	565	557	557	544	500	562	148	562		70	385	269	285	288	272	346	289	148	214
S._pyogenes	33	633	520	508	456	498	472	410	516	389	516	330		365	243	255	256	306	298	257	144	150
S._saccharolyticus	34	960	769	729	803	739	727	808	735	1018	737	976	930		192	224	225	339	327	214	299	245
S._salivarius	35	546	286	246	354	242	166	350	259	587	254	554	461	726		74	75	239	249	54	179	143
S._sanguinis	36	551	149	193	401	196	310	424	197	615	194	567	530	769	299		29	261	269	36	205	167
S._sinensis	37	549	63	225	381	238	321	402	233	613	225	566	530	777	303	164		264	274	33	200	168
S._sobrinus	38	693	572	552	537	551	557	556	553	707	554	695	645	947	541	564	569		352	259	230	234
S._suis	39	772	480	470	343	466	470	479	474	701	475	622	561	875	437	494	498	697		275	228	206
S._tigurinus	40	537	190	48	371	156	277	427	62	620	56	569	506	732	246	178	214	549	480		199	159
S._uberis	41	662	447	438	407	435	403	333	447	340	439	290	360	866	401	462	458	604	497	440		76
S._urinalis	42	636	418	404	365	374	350	262	411	494	408	434	303	796	363	411	428	566	457	406	267	

Table S10

A matrix showing the nucleotides and deletions differences between pairs of 16S-23S rRNA region sequences for *Enterococcus* species.

		1	2	3	4	5	6	7	8	9
E._avium	1		55	77	148	160	47	149	135	29
E._casseliflavus	2	238		62	115	125	28	116	102	32
E._cecorum	3	333	294		159	167	58	156	142	58
E._durans	4	332	280	416		70	115	55	35	125
E._faecium	5	336	279	416	204		123	17	37	137
E._faecalis	6	289	261	354	333	307		110	96	24
E._hirae	7	349	295	431	104	180	348		22	126
E._porcinus	8	341	271	418	114	193	336	103		112
E._raffinosus	9	62	199	307	294	295	252	309	302	

Table S11

The length of 16S-23S rRNA region, 16S rRNA gene, intergenic spacer region and 23S rRNA region for all *Streptococcus* and *Enterococcus* species.

**Species**	**16S-23S rRNA region length (bp)**	**16S rRNA gene length (bp)**	**Intergenic spacer region length (bp)**	**23S rRNA gene length (bp)**
Streptococcus acidominimus	4486	1523	468	2495
Streptococcus adjacens	4251	1525	218	2508
Streptococcus anginosus	4411	1531	387	2493
Streptococcus australis	4254	1521	240	2493
Streptococcus canis	4301	1524	283	2494
Streptococcus constellatus	4340	1531	315	2494
Streptococcus cremoris	4317	1522	300	2495
Streptococcus criceti	4649	1532	625	2492
Streptococcus cristatus	4260	1522	248	2490
Streptococcus difficilis	4295	1524	277	2494
Streptococcus downei	4564	1533	538	2493
Streptococcus durans	4366	1535	326	2505
Streptococcus dysgalactiae	4301	1524	283	2494
Streptococcus equi	4429	1524	414	2491
Streptococcus equinus	4732	1531	708	2493
Streptococcus gallolyticus	4284	1524	270	2490
Streptococcus gordonii	4267	1532	243	2492
Streptococcus infantarius	4284	1524	269	2491
Streptococcus infantis	4258	1523	242	2493
Streptococcus intermedius	4291	1531	267	2493
Streptococcus mitis	4258	1521	244	2493
Streptococcus mutans	4408	1532	384	2492
Streptococcus oligofermentans	4263	1524	249	2490
Streptococcus oralis	4256	1522	242	2492
Streptococcus ovis	4295	1533	265	2497
Streptococcus parasanguinis	4261	1523	245	2493
Streptococcus pasteurianus	4284	1524	268	2492
Streptococcus pluranimalium	4304	1534	276	2494
Streptococcus pneumoniae	4258	1521	244	2493
Streptococcus porcinus	4558	1524	538	2496
Streptococcus pseudopneumoniae	4260	1521	244	2495
Streptococcus pseudoporcinus	4498	1524	478	2496
Streptococcus pyogenes	4436	1524	418	2494
Streptococcus saccharolyticus	4263	1533	224	2506
Streptococcus salivarius	4283	1523	269	2491
Streptococcus sanguinis	4275	1523	260	2492
Streptococcus sinensis	4270	1532	248	2490
Streptococcus sobrinus	4424	1532	403	2489

**Species**	**16S-23S rRNA region length (bp)**	**16S rRNA gene length (bp)**	**Intergenic spacer region length (bp)**	**23S rRNA gene length (bp)**
Streptococcus suis	4420	1524	401	2495
Streptococcus tigurinus	4259	1524	242	2493
Streptococcus uberis	4352	1524	336	2492
Streptococcus urinalis	4292	1524	273	2495
Enterococcus avium	4273	1540	231	2502
Enterococcus casseliflavus	4266	1541	220	2505
Enterococcus cecorum	4224	1540	182	2502
Enterococcus durans	4313	1541	268	2504
Enterococcus faecium	4381	1541	335	2505
Enterococcus faecalis	4262	1542	217	2503
Enterococcus hirae	4366	1541	321	2504
Enterococcus porcinus	4346	1541	301	2504
Enterococcus raffinosus	4258	1540	215	2503

Table S12

The intraspecies polymorphism of 16S-23S rRNA region sequence within *Streptococcus* and *Enterococcus* genera[a].

Species	The lowest intraspecies nucleotide difference compared to GenBank sequences	The highest intraspecies nucleotide difference compared to GenBank sequences	Amount of complete genome sequences/genome assembly/ 16S-23S rRNA region (GenBank)
Streptococcus acidominimus	-	-	0/1
Streptococcus anginosus	0 (0%)	49 (1.11%)	6/1
Streptococcus australis	111 (2.6%)	111 (2.6%)	0/1
Streptococcus canis	3 (0.07%)	3 (0.07%)	0/1
Streptococcus constellatus	0 (0%)	18 (0.41%)	3/0
Streptococcus cremoris	0 (0%)	18 (0.42%)	14/0
Streptococcus criceti	0 (0%)	1 (0.02%)	0/0/3
Streptococcus durans	0 (0%)	73 (2.74%)	3/0
Streptococcus dysgalactiae	0 (0%)	39 (0.91%)	6/7
Streptococcus equi	0 (0%)	67 (1.51%)	6/9
Streptococcus equinus	-	-	0/4
Streptococcus gallolyticus	0 (0%)	7 (0.16%)	5/1
Streptococcus gordonii	0 (0%)	21 (0.49%)	5/3
Streptococcus infantarius	0 (0%)	40 (0.93%)	2/0
Streptococcus intermedius	0 (0%)	20 (0.47%)	6/1
Streptococcus mitis	0 (0%)	37 (0.87%)	5/0
Streptococcus mutans	0 (0%)	20 (0.45%)	9/2/3
Streptococcus oligofermentans	0 (0%)	4 (0.09%)	1/2
Streptococcus oralis	0 (0%)	55 (1.29%)	4/1
Streptococcus parasanguinis	0 (0%)	40 (0.94%)	2/0
Streptococcus pasteurianus	0 (0%)	2 (0.05%)	2/1
Streptococcus pluranimalium	0 (0%)	16 (0.37%)	2/0
Streptococcus pneumoniae	0 (0%)	496 (11.65%)	52/83
Streptococcus porcinus	0 (0%)	0 (0%)	0/1
Streptococcus pseudopneumoniae	16 (0.4%)	16 (0.4%)	1/0
Streptococcus pseudoporcinus	0 (0%)	0 (0%)	0/1
Streptococcus pyogenes	0 (0%)	98 (2.2%)	136/65
Streptococcus salivarius	0 (0%)	33 (0.77%)	9/2
Streptococcus sanguinis	7 (0.16%)	31 (0.72%)	1/5
Streptococcus sobrinus	0 (0%)	15 (0.34%)	4/2
Streptococcus suis	0 (0%)	58 (1.31%)	43/2
Streptococcus tigurinus	38 (0.9%)	38 (0.9%)	1/0
Streptococcus uberis	0 (0%)	5 (0.11%)	2/2
Streptococcus urinalis	0 (0%)	0 (0%)	0/1
Enterococcus casseliflavus	1 (0.02%)	4 (0.09%)	2/0
Enterococcus cecorum	1 (0.02%)	28 (0.66%)	6/1

**Species**	**The lowest intraspecies nucleotide difference compared to GenBank sequences**	**The highest intraspecies nucleotide difference compared to GenBank sequences**	**Amount of complete genome sequences/genome assembly/ 16S-23S rRNA region (GenBank)**
Enterococcus durans	0 (0%)	42 (0.97%)	3/0
Enterococcus faecium	0 (0%)	102 (2.33%)	70/58
Enterococcus faecalis	0 (0%)	17 (0.4%)	31/1
Enterococcus hirae	0 (0%)	71 (2.67%)	3/1

[a] For species not included in a Table (*Streptococcus adjacens*, *Streptococcus cristatus, Streptococcus difficilis, Streptococcus downei, Streptococcus infantis, Streptococcus ovis, Streptococcus saccharolyticus*, *Streptococcus sinensis, Enterococcus avium, Enterococcus porcinus*, *Enterococcus raffinosus*), there are no reference genomes available.

Figure S1

The comparison between phylogenetic trees based on 16S-23S rRNA region and 16S rRNA, *rpoB*, *sodA* and *tuf* genes for both *Streptococcus* and *Enterococcus* species.

The thicker branches reflect a low topological score and result from different positions on the compared trees.

Streptococcus

16S rRNA gene vs 16S-23S rRNA region

Overall topological score = 72.4 %

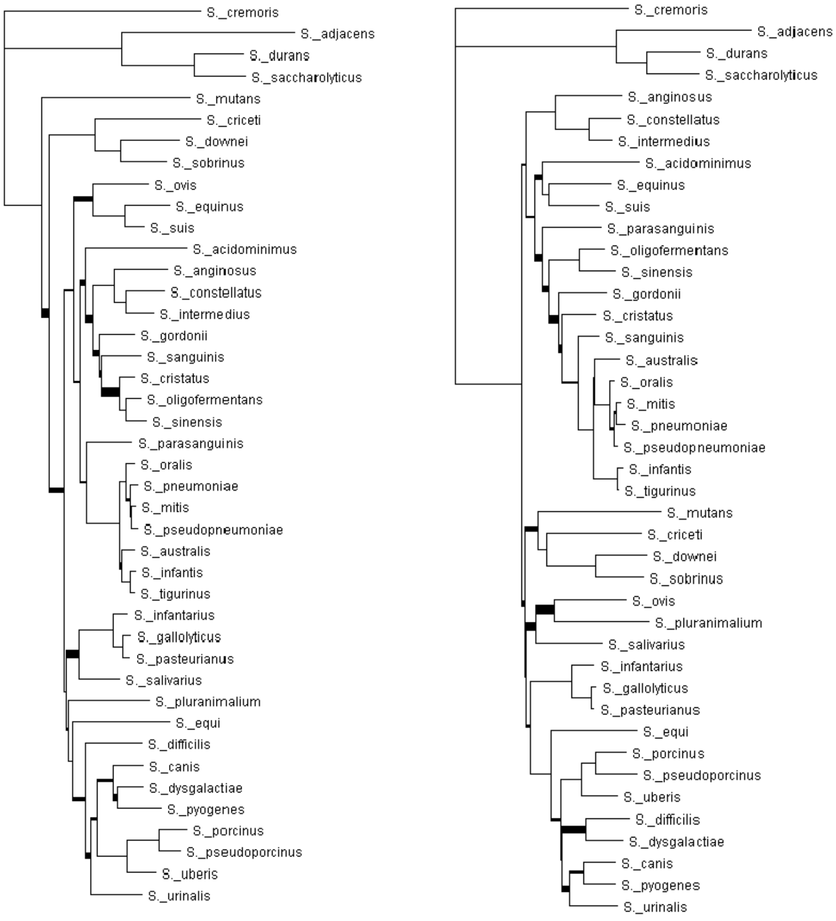

rpoB gene vs 16S-23S rRNA region

Overall topological score = 71.6 %

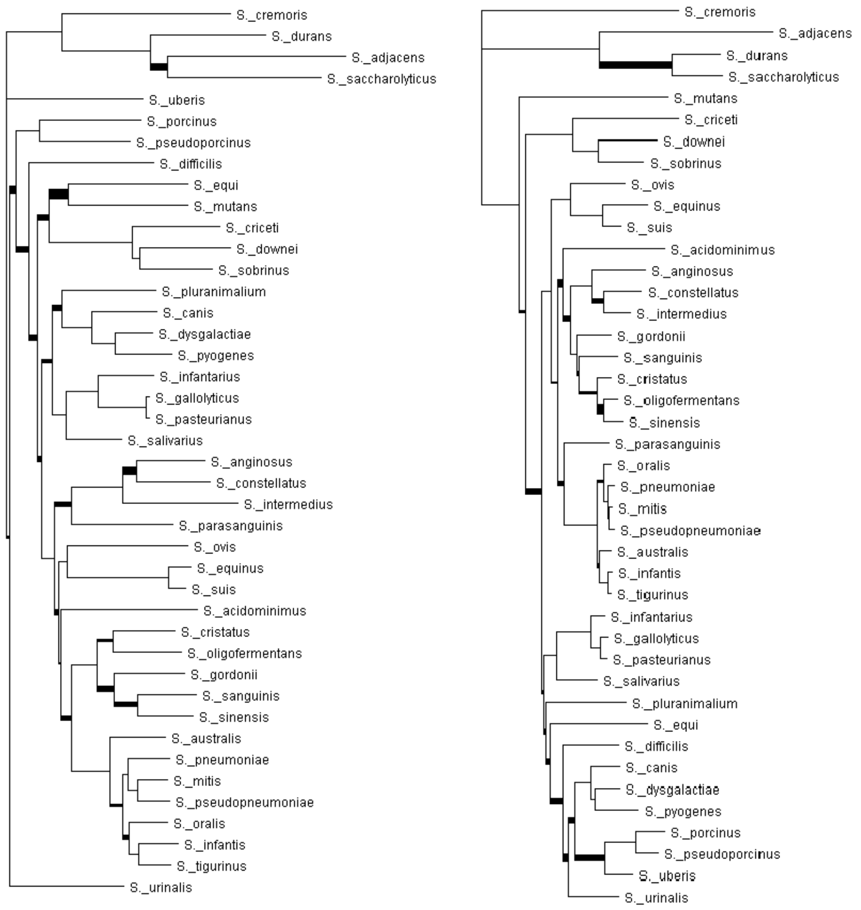

sodA gene vs 16S-23S rRNA region

Overall topological score = 70.4 %

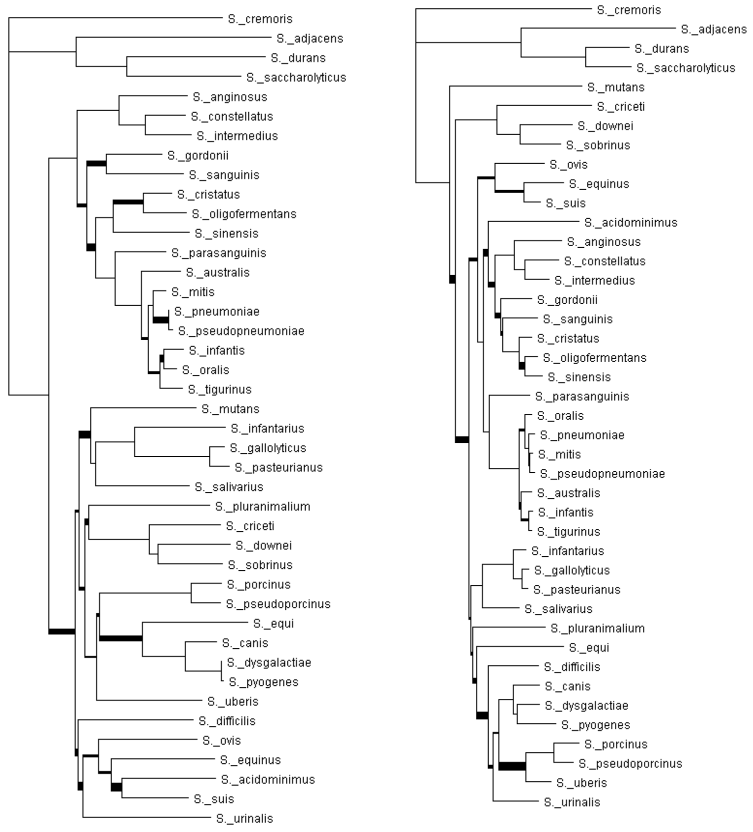

tuf gene vs 16S-23S rRNA region

Overall topological score = 70 %

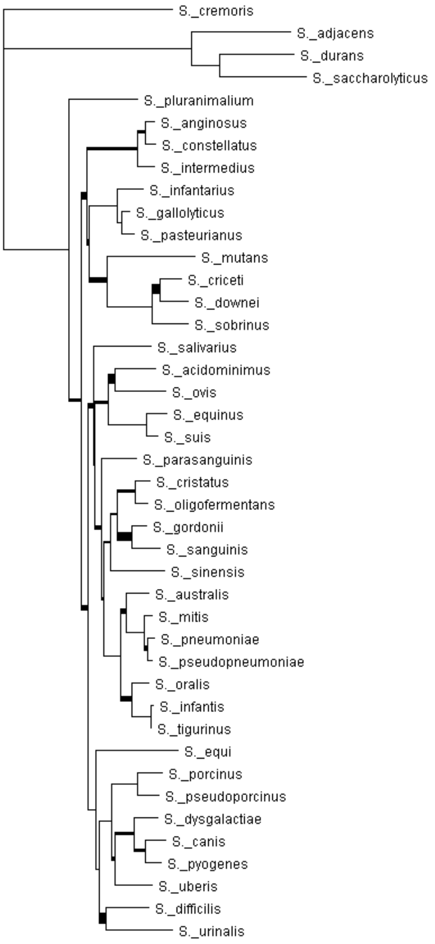

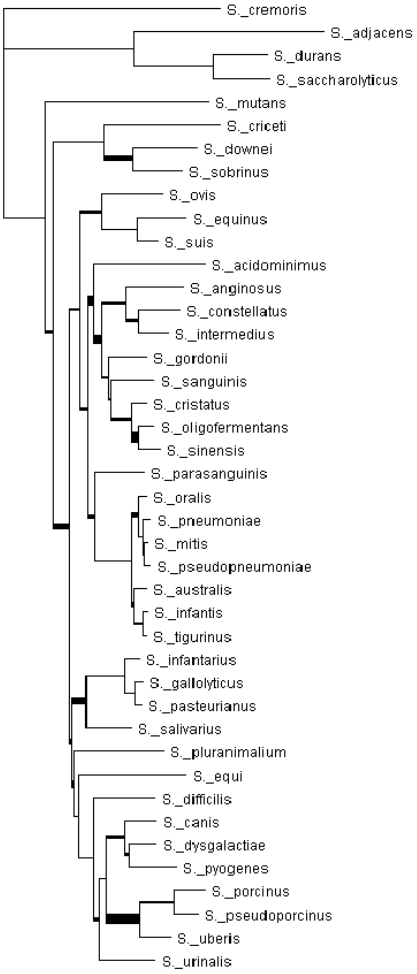

Enterococcus

16S rRNA gene vs 16S-23S rRNA region

Overall topological score = 76.7 %

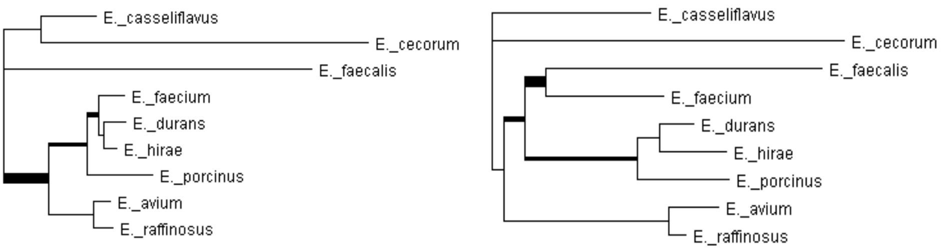

rpoB gene vs 16S-23S rRNA region

Overall topological score = 62.5 %

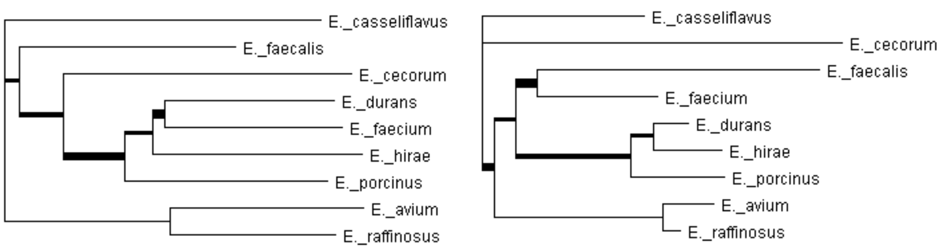

sodA gene vs 16S-23S rRNA region

Overall topological score = 75.6 %

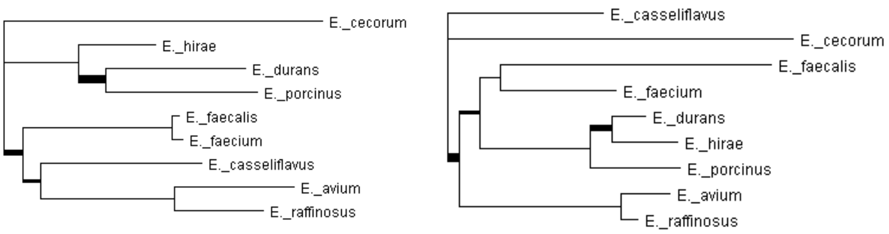

tuf gene vs 16S-23S rRNA region

Overall topological score = 66.4 %

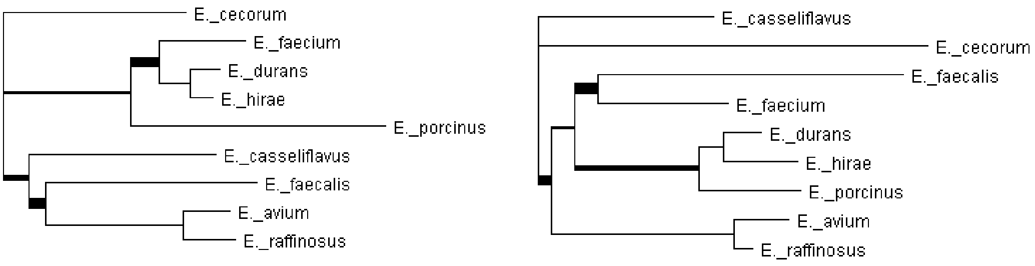

Streptococcus

16S rRNA gene vs *sodA* gene

Overall topological score = 66.5 %

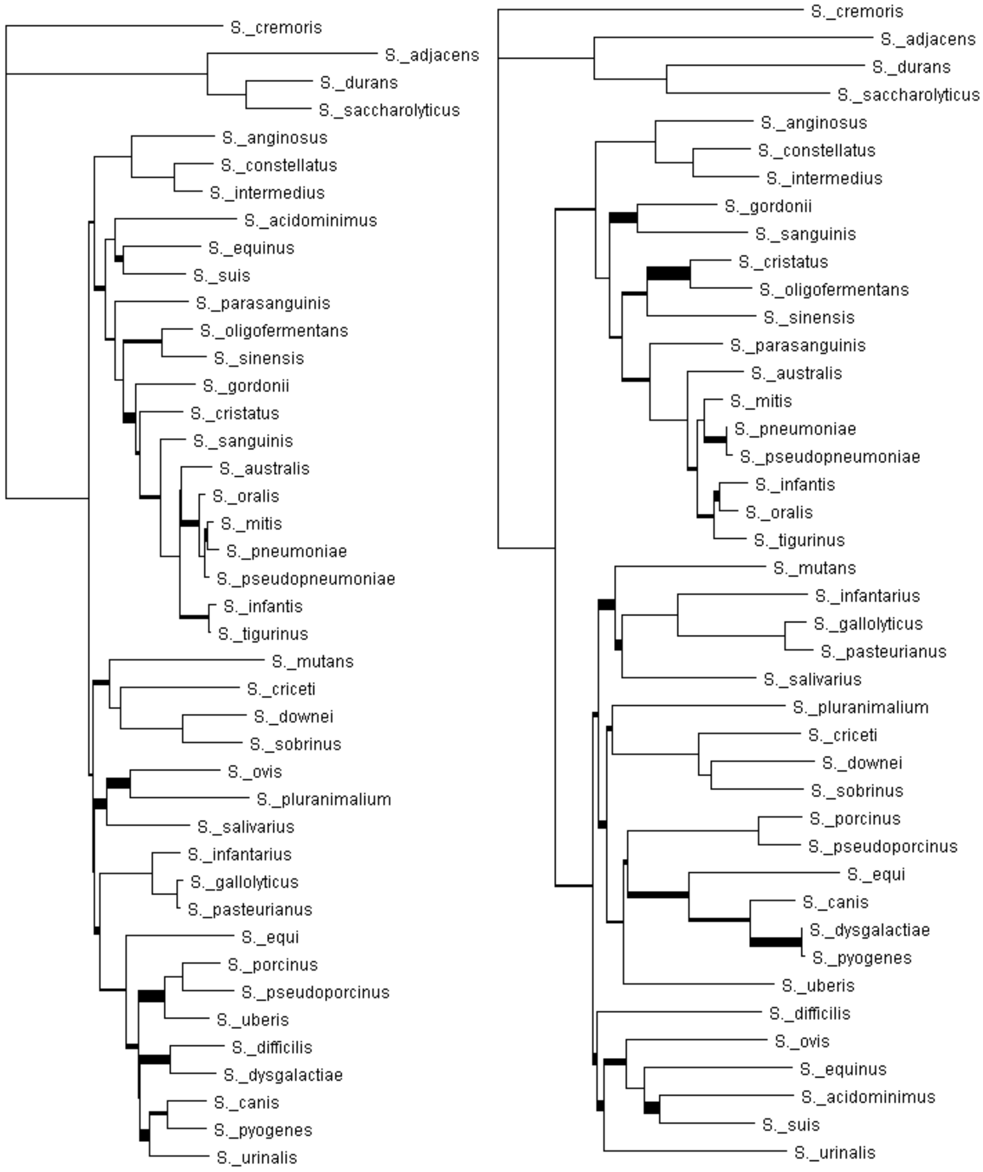

Streptococcus

16S rRNA gene vs *rpoB* gene

Overall topological score = 61.7 %

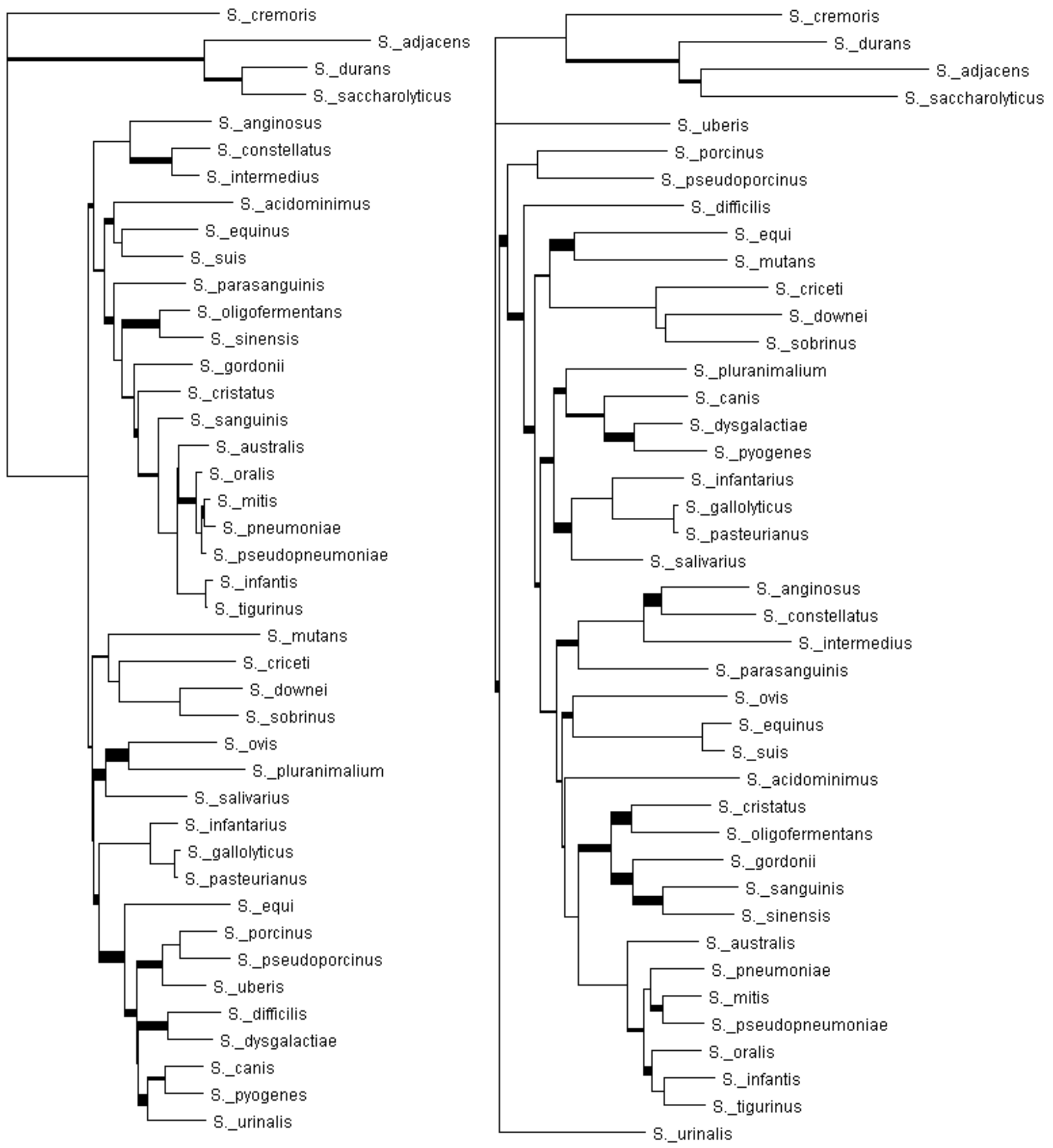

Streptococcus

16S rRNA gene vs *tuf* gene

Overall topological score = 69.1 %

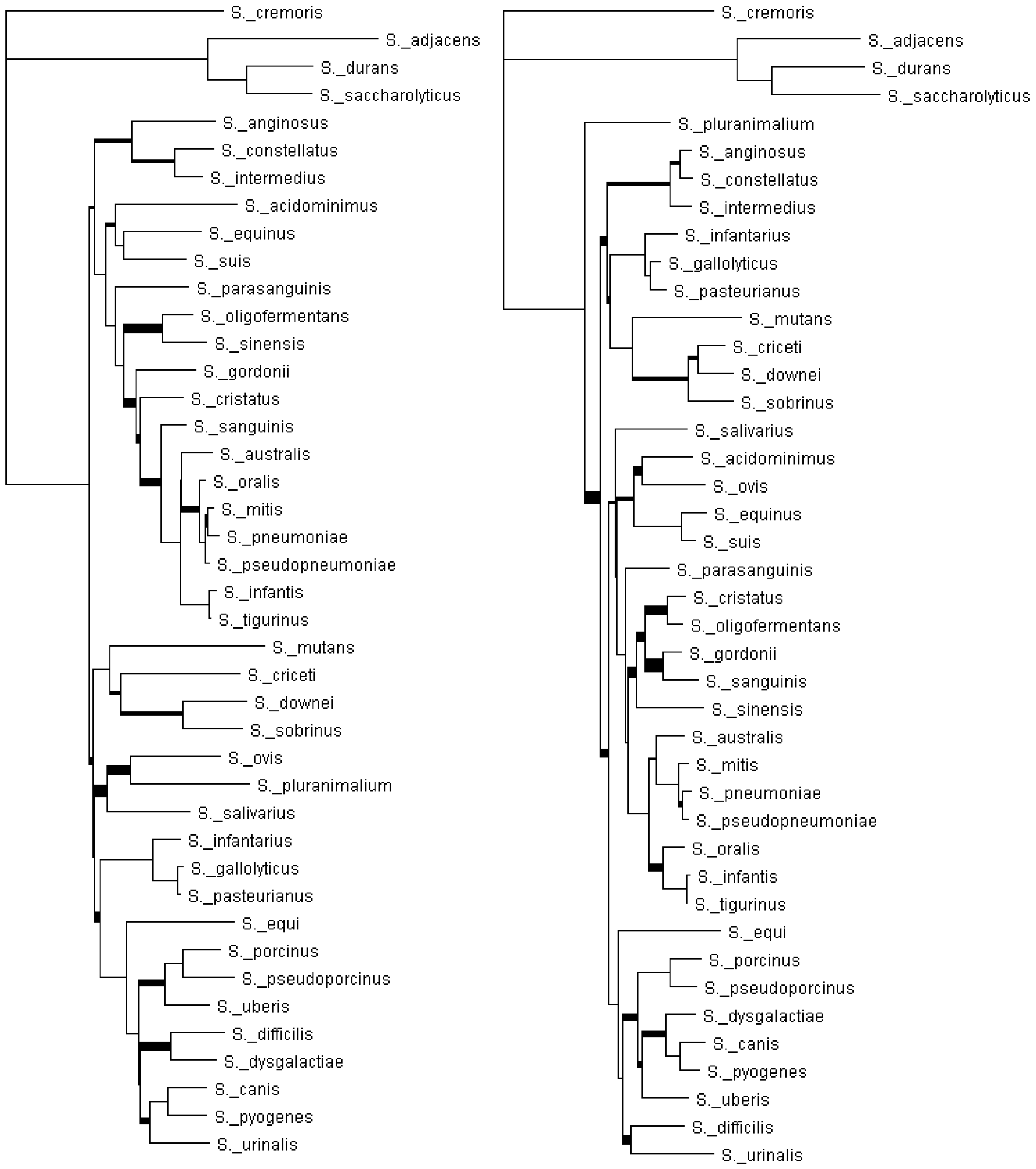

Enterococcus

16S rRNA gene vs *sodA* gene

Overall topological score = 56.4 %

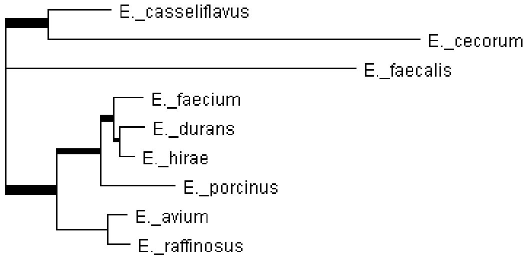

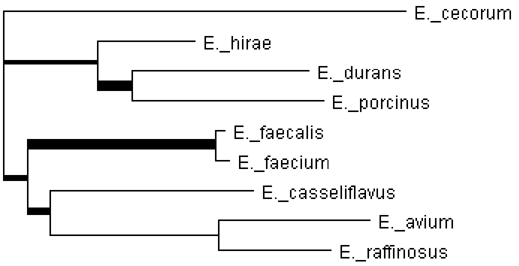

Enterococcus

16S rRNA gene vs *rpoB* gene

Overall topological score = 67.0 %

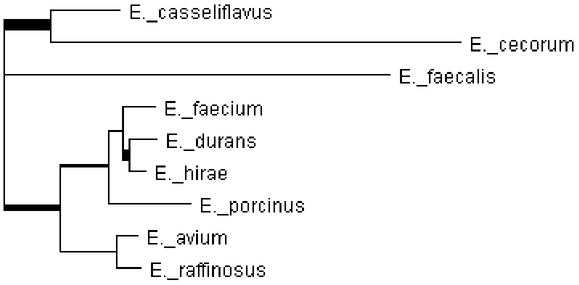

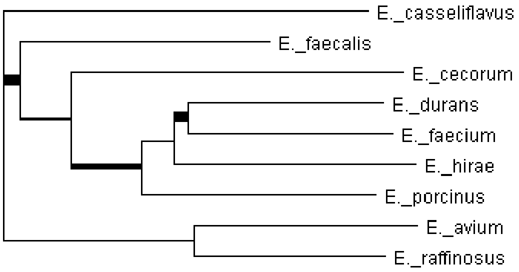

Enterococcus

16S rRNA gene vs *tuf* gene

Overall topological score = 78.9 %

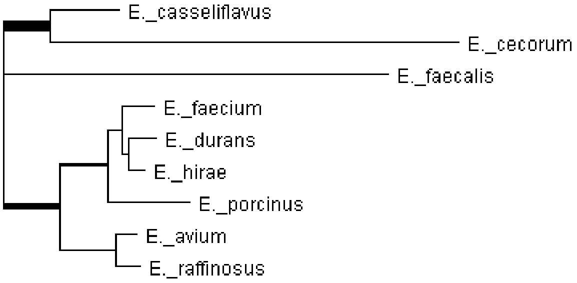

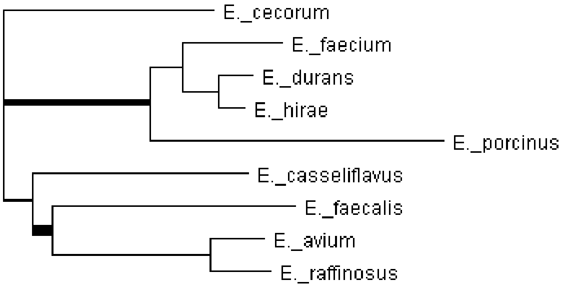

Streptococcus

tuf gene vs *sodA* gene

Overall topological score = 70.4 %

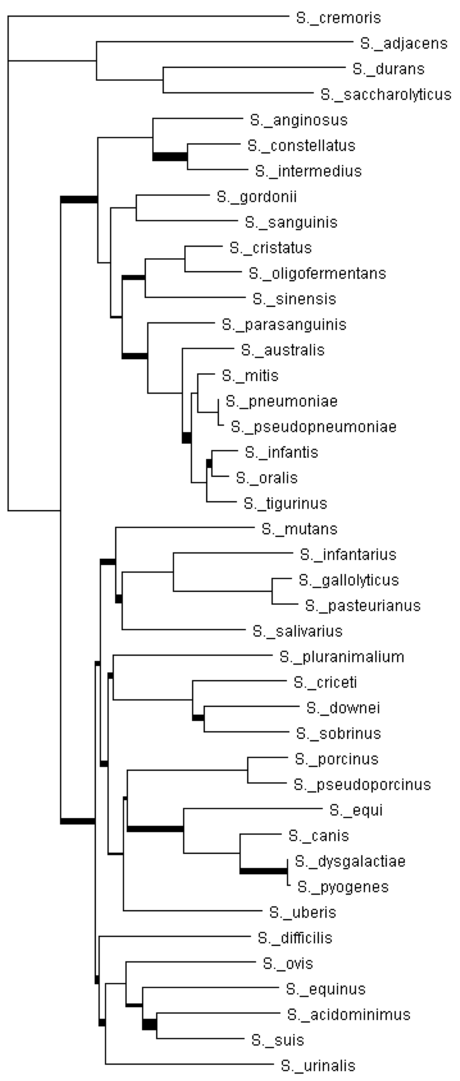

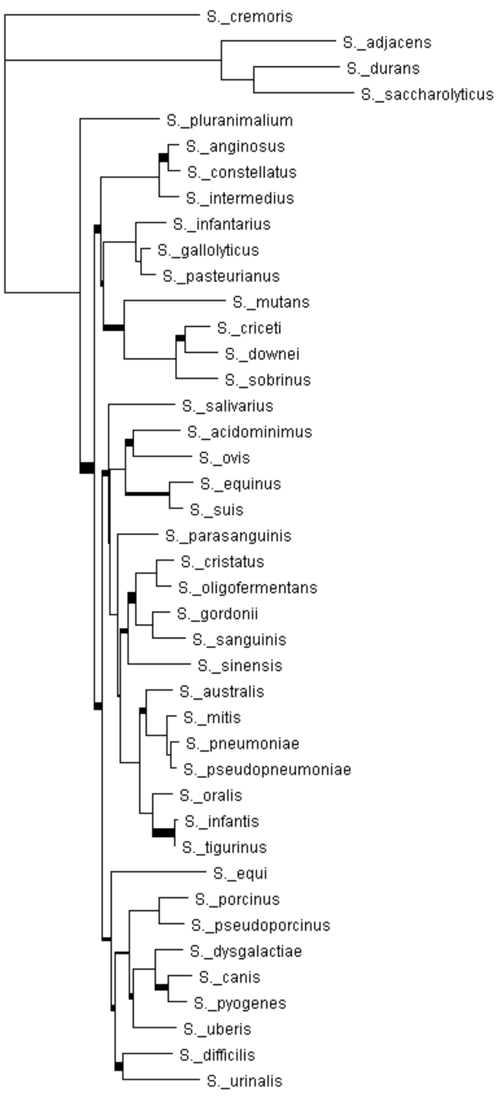

Streptococcus

rpoB gene vs *tuf* gene

Overall topological score = 70.3 %

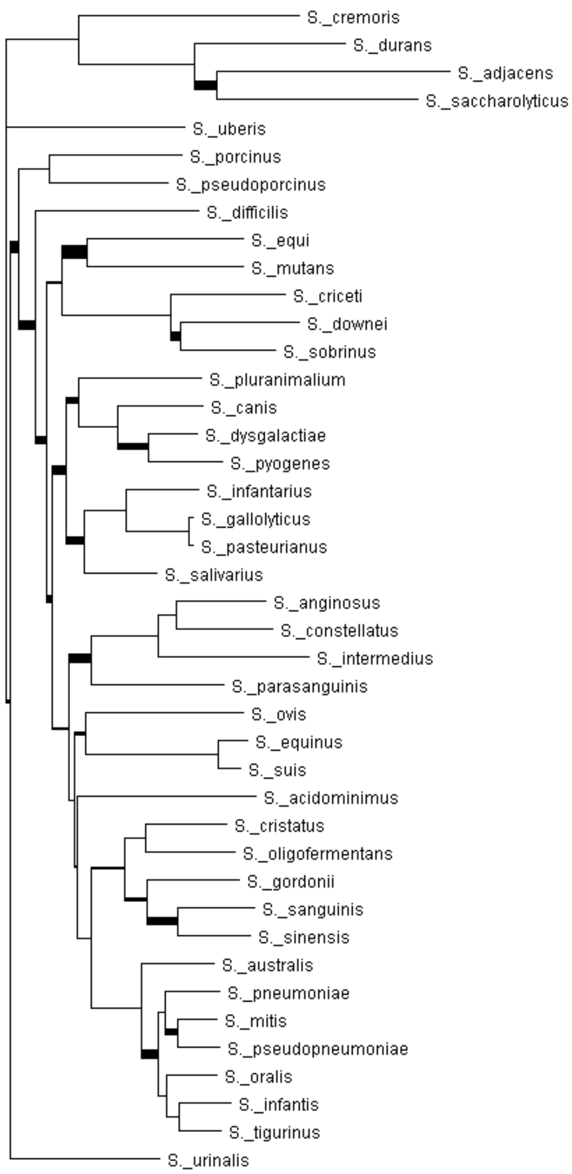

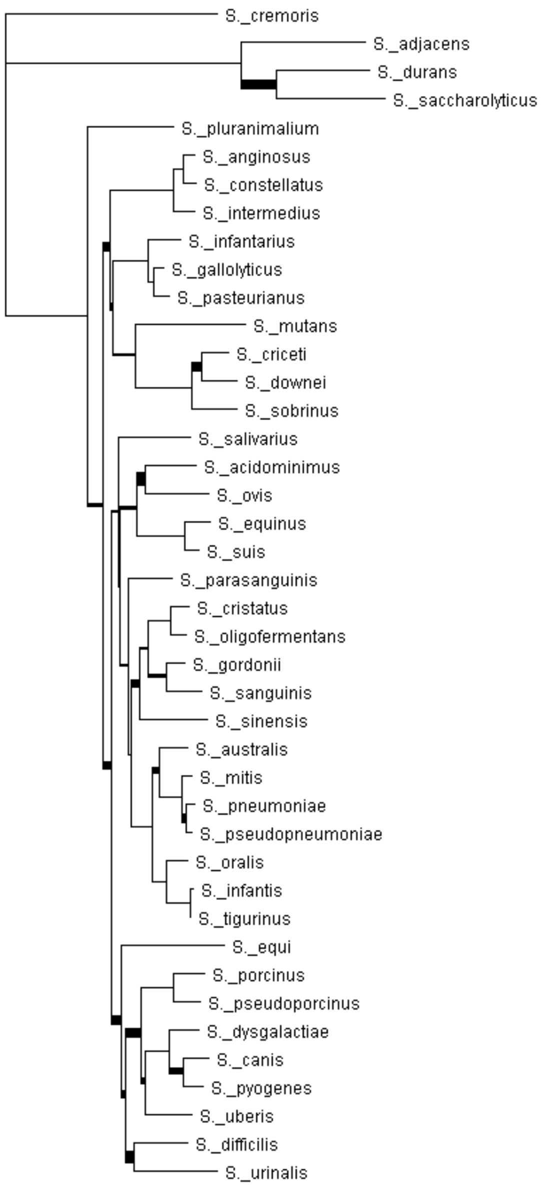

Streptococcus

rpoB gene vs *sodA* gene

Overall topological score = 67.3 %

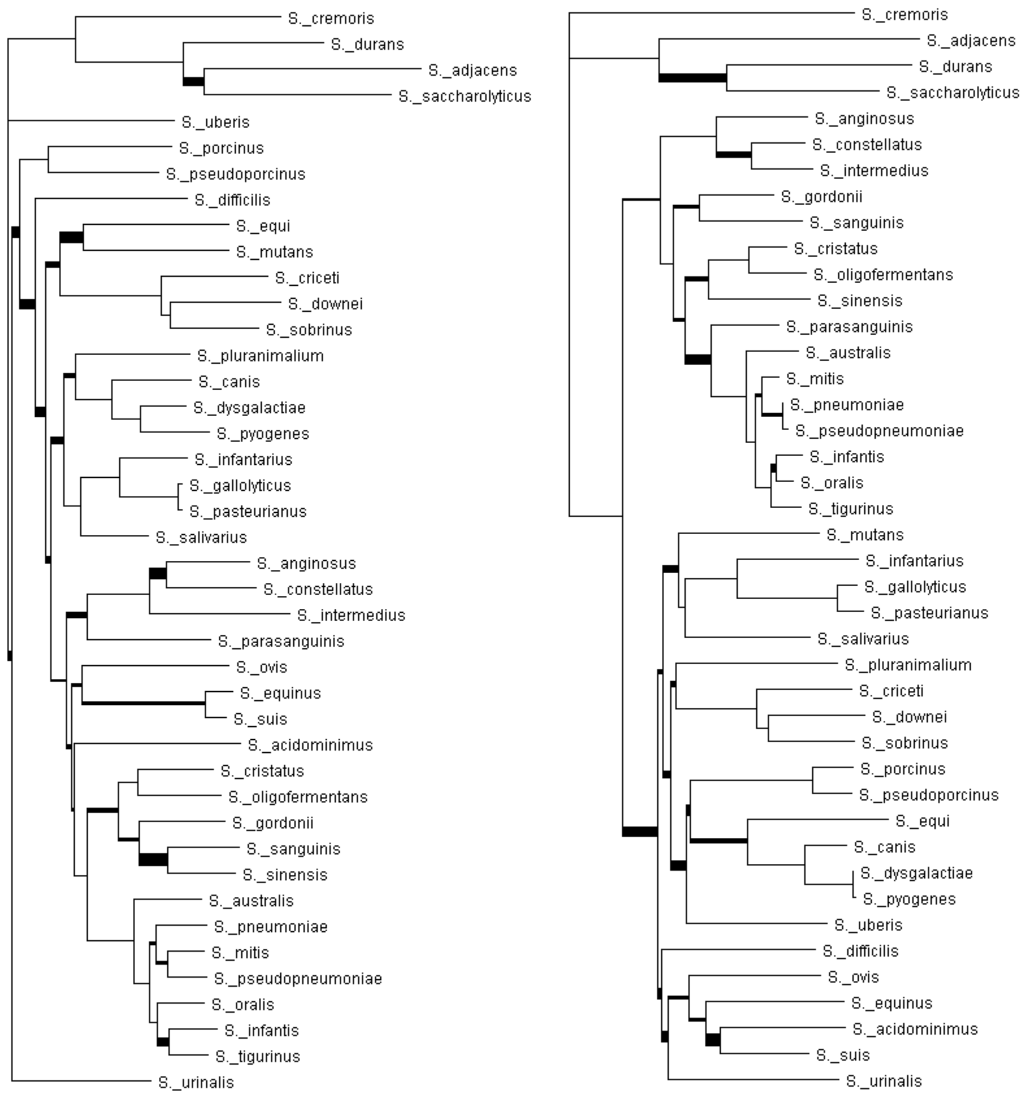

Enterococcus

tuf gene vs *sodA* gene

Overall topological score = 62.8 %

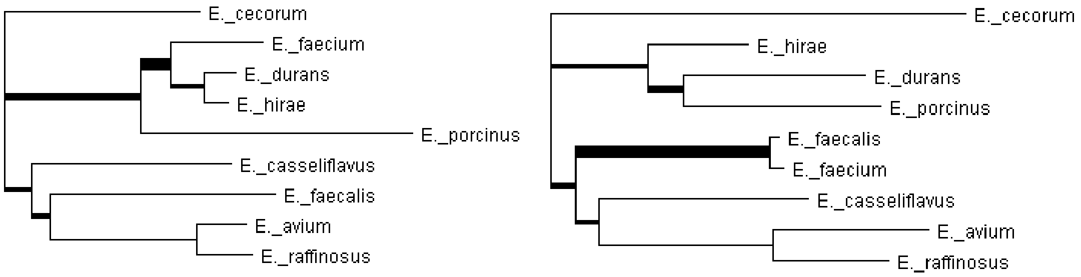

Enterococcus

rpoB gene vs *tuf* gene

Overall topological score = 80.6 %

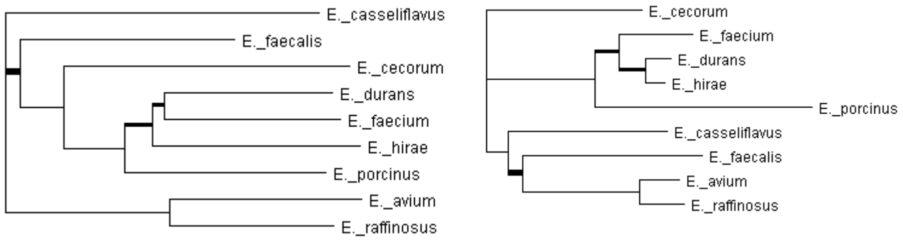

Enterococcus

rpoB gene vs *sodA* gene

Overall topological score = 68.9 %

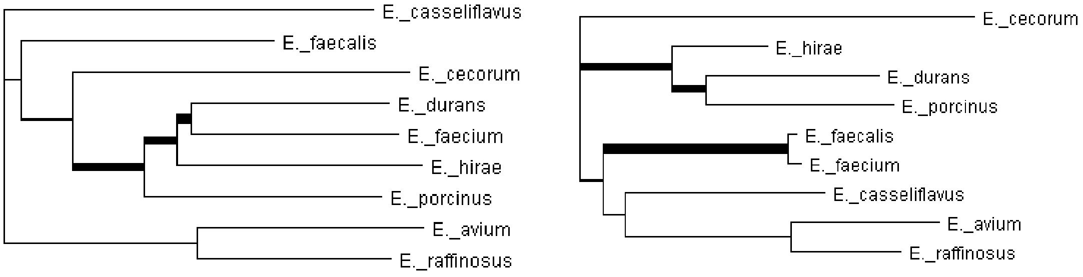

Supplement: Supplementary file 1 — Additional file 1. Includes (i) matrixes with differences in the number of nucleotides and deletions between sequence pairs of all Sanger-based and NGS-based method, (ii) table with the length of 16S–23S rRNA region, 16S rRNA gene, intergenic spacer region and 23S rRNA region for all species, (iii) table with the intraspecies polymorphism of 16S–23S rRNA region sequence within Streptococcus and Enterococcus genera, (iv) the comparison between phylogenetic trees based on 16S–23S rRNA region and 16S rRNA, rpoB, sodA and tuf genes for both Streptococcus and Enterococcus species. [file 13756_2019_622_MOESM1_ESM.pdf]
